# TDP‐43 proteinopathies and neurodegeneration: insights from *Caenorhabditis elegans* models

**DOI:** 10.1111/febs.70239

**Published:** 2025-09-02

**Authors:** Ghulam Jeelani Pir, Joerg Buddenkotte, Majid Ali Alam, Ahmed Own, Randall J. Eck, Brian C. Kraemer, Eckhard Mandelkow, Martin Steinhoff

**Affiliations:** ^1^ The Neuroscience Institute, Academic Health System Hamad Medical Corporation Doha Qatar; ^2^ Translational Research Institute, Academic Health System Hamad Medical Corporation Doha Qatar; ^3^ Dermatology Institute, Academic Health System Hamad Medical Corporation Doha Qatar; ^4^ Department of Dermatology and Venereology, Rumailah Hospital Hamad Medical Corporation Doha Qatar; ^5^ Geriatrics Research Education and Clinical Center (GRECC) Veterans Affairs Puget Sound Healthcare System Seattle WA USA; ^6^ Division of Gerontology and Geriatric Medicine, Department of Medicine University of Washington Harborview Medical Center Seattle WA USA; ^7^ Department of Psychiatry and Behavioral Sciences University of Washington Seattle WA USA; ^8^ Department of Laboratory Medicine and Pathology University of Washington Seattle WA USA; ^9^ German Center for Neurodegenerative Diseases (DZNE) Bonn Germany; ^10^ Department of Gerontopsychiatry and Neurodegenerative Diseases UKB, University of Bonn Medical School Bonn Germany; ^11^ Department of Medicine Weill Cornell Medicine Qatar, Qatar Foundation‐Education City Doha Qatar; ^12^ College of Medicine Qatar University Doha Qatar; ^13^ Department of Medicine Weill Cornell Medicine New York NY USA

**Keywords:** acetylcholine, Alzheimer's disease (AD), amyotrophic lateral sclerosis (ALS), *C. elegans*, extracellular vesicles (EV), frontotemporal dementia (FTD), GABA, G‐protein coupled receptors, Huntington's disease, ion channels, limbic‐predominant age‐related TDP‐43 encephalopathy (LATE), Parkinson's disease (PD), proteinopathies, tau, TDP‐43/TDP‐1

## Abstract

TDP‐linked proteinopathies, including amyotrophic lateral sclerosis (ALS), frontotemporal dementia (FTD) and limbic‐predominant age‐related TDP‐43 encephalopathy (LATE), are characterised by pathogenic deposits containing transactive response DNA‐binding protein 43 (TDP‐43) in the brain and spinal cord of patients. These hallmark pathological features are associated with widespread neuronal dysfunction and progressive neurodegeneration. TDP‐43's role as an essential RNA/DNA‐binding protein in RNA metabolism and gene expression regulation is clear, but deciphering the intricate pathophysiological mechanisms underpinning TDP‐43‐mediated neurodegeneration is paramount for developing effective therapies and novel diagnostic tools for early detection before frank neuronal loss occurs. The nematode *Caenorhabditis elegans*, with highly conserved TDP‐43 orthologue TDP‐1, serves as a powerful genetic model to investigate the molecular underpinnings of TDP‐43 proteinopathies. Here, we provide a brief overview of the structural and functional characteristics of TDP‐43 and TDP‐1, highlighting their conserved roles in RNA metabolism, stress responses, and neurodegeneration. We then delve into the pathobiology of TDP‐43, drawing insights from *C. elegans* models expressing either monogenic TDP‐43 variants or bigenic combinations with ALS‐associated risk genes, and discuss how these models have advanced our understanding of the pathomechanisms of TDP‐43 proteinopathies. By employing its simplicity and genetic manipulability, we discuss how these models have helped identify chemical and genetic suppressors of TDP‐43‐induced phenotypes, including small molecules like Pimozide and the probiotic *Lacticaseibacillus rhamnosus* HA‐114, now in clinical trials. This review underscores the translational value of *C. elegans* in unraveling the biochemical pathways and interactions in TDP‐43 proteinopathies that perturb cellular physiology, potentially facilitating mechanism‐based therapy development.

## Graphical abstract

The manuscript explores structural and functional features of TDP‐43 and its worm homologue, TDP‐1, highlighting conserved and divergent structural and functional features. Using genetically engineered *C. elegans* models, key pathological features of TDP‐43 proteinopathies—including aggregation, neurodegeneration, and motor deficits—are recapitulated, enabling high‐throughput pharmacological and genetic screening and mechanistic studies, making them powerful tools for uncovering disease pathways in ageing and neurodegeneration.

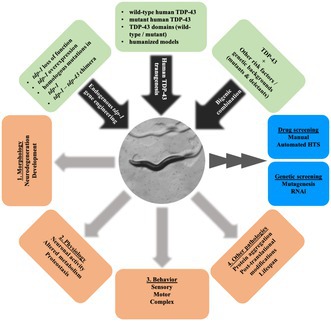

**Abbreviations**
ALS: amyotrophic lateral sclerosis
ALS‐FTDSD: Amyotrophic Lateral Sclerosis‐Frontotemporal Dementia Spectrum Disorder
ANG: angiogenin
APA: alternative polyadenylation
C9ORF72: chromosome 9 open reading frame 72
CFTR: cystic fibrosis transmembrane conductance regulator
CTD: C‐terminal domain
ERAD: endoplasmic reticulum‐associated degradation
EV: extracellular vesicles
FTD: frontotemporal dementia
FTLD: frontotemporal lobar degeneration
FUS: fused in sarcoma
GABA: gamma‐aminobutyric acid
GRN: progranulin
HIV‐1: human immunodeficiency virus type 1
HSF‐1: Heat Shock Factor‐1
LATE: Limbic‐predominant Age‐related TDP‐43 Encephalopathy
MAPT: microtubule associated protein tau
MSP: multisystem proteinopathy
NES: nuclear export signal
NLS: nuclear localisation signal
NMJs: neuromuscular junctions
NTD: N‐terminal domain
PROTACs: proteolysis targeting chimeras
RAD‐23: radiation sensitivity abnormal‐23
RRM: RNA recognition motifs
SOD‐1: superoxide dismutase
STMN2: Stathmin 2
SUMO: SUMOylation
TARDBD: transactive response DNA‐binding protein of 43 kDa
TIM22: translocase of inner mitochondrial membrane protein 22
TIR‐1: Toll Interleukin 1 Receptor domain adaptor protein
TOM70: translocase of outer mitochondrial membrane protein 70
TTBK: tau tubulin kinase
UBQLN2: ubiquilin‐2
UPS: ubiquitin proteasome system
WT: wild type
XPO1: exportin

## Introduction

Human transactive response DNA‐binding protein of 43 kDa (TDP‐43) is a ubiquitously expressed RNA/DNA binding protein belonging to the heterogeneous nuclear ribonucleoprotein (hnRNP) family that is highly conserved in human, mouse, *Drosophila melanogaster* and *Caenorhabditis elegans* [1]. Initially identified in 1995 as a transcriptional repressor of human immunodeficiency virus type 1 (HIV‐1) through its binding to chromosomally integrated TAR regulatory element of HIV‐1 [2], TDP‐43 was later reported to also repress mouse *Sp‐10* [3] and human cyclin‐dependent kinase 6 (*CDK6*) genes [4]. Further studies reported its involvement in regulating the alternate splicing of cystic fibrosis transmembrane conductance regulator (*CFTR*) [5, 6, 7]. TDP‐43, however, gained prominence in 2006 when it was identified as the main constituent protein of the ubiquitinated inclusions observed in the neurons and glial cells of sporadic amyotrophic lateral sclerosis (ALS) patients and in patients with frontotemporal lobar degeneration with ubiquitin‐positive inclusions (formerly FTLD‐U, now classified as FTLD‐TDP) [8, 9, 10, 11, 12], a common neuropathological subtype of frontotemporal dementia (FTD). The discovery marked a pivotal moment in ALS research, fundamentally altering our understanding of disease pathogenesis. Both ALS and FTD are late‐onset progressive neurodegenerative diseases that exhibit a substantial overlap in genetic, clinical, and neuropathological features. However, the two are separate disorders affecting distinct regions of the nervous system.

ALS, also known as Charcot's disease or Lou Gehrig's disease, is the most common adult‐onset motor neuron disease with a worldwide prevalence of ∼5 individuals per 100,000 each year. The disease primarily affects upper and lower motor neurons of the brain and spinal cord, leading to progressive muscle weakness, paralysis, and ultimately death due to respiratory failure [13, 14]. While only 10% of the ALS cases are familial (fALS), with hexanucleotide repeat expansion in *C9ORF72* gene representing the most common forms [15, 16, 17], the majority account for sporadic cases (sALS) with no known cause or familial history [18]. Yet, mutations in genes typically associated with familial ALS, such as *ANG* [19], *FUS* [20], *SOD1* [21], *TARDBP* [22] may be detected in patients with sporadic ALS [19, 23, 24, 25], indicating some genetic influence [26]. Nonetheless, the two forms are clinically indistinguishable with an average disease onset at ∼50 years [27, 28], notable exceptions are patients with mutations in the ALS‐associated genes such as the *SOD1*‐A4V variant [29], or the *FUS*c.1574C>T (P525L) and *c.1554_1557*delACAG mutations [30] that show distinct phenotypes and an early onset. Similarly, patients with relatively common *C9ORF72* hexanucleotide repeat expansion have a characteristic phenotype and pathological features distinct from the other ALS forms [31, 32].

FTD is a clinical syndrome that primarily affects the frontal and temporal lobes of the brain [10, 33], leading to a spectrum of personality, behavioural and psychiatric alterations along with progressive deterioration in language abilities. In stark contrast to ALS, which seldom associates with dementia, FTD ranks among the most common causes of dementia, second only to Alzheimer's disease, particularly in individuals younger than 65 years of age, and has a worldwide yearly estimated prevalence of 15–22 cases per 100,000 people [34, 35]. FTD is largely familial (30–50%) and inherited in an autosomal dominant fashion, with mutations in the microtubule‐associated protein tau (*MAPT*), progranulin (*GRN*), and *C9ORF72* genes representing the most common forms [36, 37]. Although sporadic cases do not have a known cause, a history of head trauma is thought to elevate the risk of developing FTD [38, 39].

While FTD is marked by a notable initial focal tissue loss in the frontal and/or temporal lobes, which gradually extends over time to encompass significant areas of the brain [40], ALS in contrast shows brain tissue degeneration, especially motor cortex atrophy, only in a minority of the patients [41]. Irrespective of sporadic or familial forms, protein inclusions composed of hyperphosphorylated, ubiquitinated and N‐terminally truncated TDP‐43 [42, 43, 44] are a hallmark of the bulk of ALS patients [11, 45] and nearly 50% of all FTLD cases [46, 47]. Moreover, the regional distribution of TDP‐43 neuropathology in both ALS and FTLD shows a significant clinical overlap and mutations in the TDP‐43‐encoding gene can result in both the conditions [45].

Both ALS and FTLD‐TDP share this common pathological hallmark, i.e. mislocalisation and aggregation of TDP‐43, with yet another recently defined condition termed Limbic‐predominant Age‐related TDP‐43 Encephalopathy (LATE). TDP‐43 inclusions in LATE primarily affect the limbic system, contributing to age‐related amnestic dementia that clinically mimics Alzheimer's disease but occurs without significant amyloid‐beta pathology [48]. Interestingly, LATE is gaining recognition as a significant co‐pathology in Alzheimer's disease (AD), profoundly impacting disease progression and clinical outcomes, with 30–50% of AD cases showing TDP‐43 pathology, particularly among older individuals [48]. LATE co‐pathology in AD is marked by accelerated cognitive decline, greater memory impairment, and increased hippocampal atrophy compared to AD cases without TDP‐43 pathology [49, 50]. Also, LATE in AD exacerbates neurodegeneration, thereby contributing to a faster progression from mild cognitive impairment to dementia [51]. This overlap further complicates AD diagnosis and distinction between the two conditions [52]. Consequently, ALS, FTLD and LATE along with two other diseases that also show TDP‐43 inclusions in relation to primary lateral sclerosis and progressive muscular atrophy, are collectively referred to as TDP‐proteinopathies [33, 53]. Across these disorders, TDP‐43 is central to neurodegeneration and, impaired RNA metabolism, disrupted proteostasis, and neuroinflammation are seen as common underlying mechanisms [54]. Understanding the molecular pathways driving TDP‐43 pathology in these diseases and the identification of biomarkers for precise clinical diagnosis could lead to targeted therapeutic strategies that benefit multiple conditions to improve patient outcomes.

Considering this, circulating TDP‐43 and its phosphorylated and aggregated forms in plasma and cerebrospinal fluid (CSF) have been investigated as potential diagnostic biomarkers for ALS and FTLD [55, 56, 57, 58, 59]. Although earlier findings have been inconsistent, they address a critical gap that hinders emerging therapies aimed at early‐stage intervention when neuronal loss is less advanced. The lack of specificity [60] and sensitivity in detecting circulating TDP‐43 levels in early‐stage ALS or atypical cases [61], that limited its diagnostic value particularly in distinguishing between ALS and other TDP‐43 proteinopathies, have been largely addressed by: (a) recent technological advancements such as single‐molecule array (Simoa) and immunoprecipitation‐mass spectrometry (IP‐MS), which enable enhanced detection and more reliable quantification, and (b) combining more than one biomarker. As an example, a recent study demonstrated that a combination of TDP‐43 levels and 3‐ to 4‐repeat tau ratios (3R/4R) in the plasma extracellular vesicles (EVs) may inform the diagnosis of FTD, FTD spectrum disorders and ALS [62]. Hence, the investigation of multiple biomarkers may enhance our understanding of molecular mechanisms as well as the accuracy of diagnosis and help in identifying cases with overlapping pathologies.

Overall, molecular pathways implicated in TDP‐43 proteinopathies and complex interactions between factors involved need to be elucidated for any substantial progress in therapeutic intervention at early stages where neurodegeneration can be curbed. Simple non‐mammalian eukaryotic models like *C. elegans* have been instrumental in revealing key mechanistic details involved in the pathogenesis of these diseases. Originally introduced as a genetic model by Sydney Brenner in the 1960s, *C. elegans* is a small (1.0–1.5 mm), bisexual nematode with a life cycle of approx. three weeks, a short generation time of approx. three days, a large brood size of approx. 300 per hermaphrodite through self‐fertilisation or approx. 1000 offsprings through cross‐fertilisation with males, and a transparent body allowing visualisation of internal structures [63]. The nematode's development (from zygote to adult) has been comprehensively mapped, comprising an invariant number of 959 and 1031 somatic cells, including 302 and 391 neurons in hermaphrodites and males, respectively [64]. *C. elegans* remains the only organism with a fully mapped nervous system, featuring approximately 5000 synapses, 2000 neuromuscular junctions and 600 gap junctions [65] and was the first with a sequenced genome harbouring approx. 19,700 coding sequences and approx. 1,300 non‐coding RNAs [66]. It is estimated that around 60% of human genes have homologues in *C. elegans* [67, 68, 69] and many biochemical pathways are likely to be conserved due to preserved protein functions (e.g. [70]), reflecting high genetic and functional conservation. Similarly, conserved neuronal functions that give rise to complex behaviours such as chemo‐ and mechanosensation, avoidance of noxious stimuli and thermotaxis, and major neurotransmitter systems, i.e. cholinergic, dopaminergic, GABAergic, glutamatergic and serotonergic are represented, making this nematode ideal for modeling neurodegenerative diseases. In this review, we will discuss how this simple model organism with its robust genetic toolkit and high genetic and functional homology to humans, can be a fruitful starting point to enhance our understanding of neurodegenerative disease processes related to TDP‐43 proteinopathies.

## Main

### Structural features of human TDP‐43

TAR DNA‐binding protein is encoded by the TAR DNA‐binding protein gene, *TARDBP*, which consists of six exons and four introns located on chromosome 1p36.22 [71]. Multiple isoforms are predicted to be generated through alternative splicing of the *TARDBP* gene, the canonical form and the most studied, being 414 amino acids long TARDBP of 43 kDa (TDP‐43) [1, 72, 73]. TDP‐43 is ubiquitously expressed and predominantly nuclear, but can be detected at low levels in the cytoplasm due to constant nucleocytoplasmic shuttling [74] and in mitochondria [75, 76], where it exerts its physiological functions. In TDP‐43 proteinopathies, however, protein inclusions containing post‐translationally modified TDP‐43 accumulate in the cytoplasm [11, 77, 78]. The caveat is that a mere cytosolic mislocalisation may not be sufficient for TDP‐43 pathology [79].

**Fig. 1:**
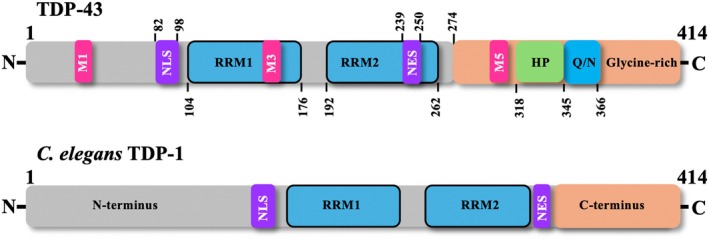
TAR DNA‐binding protein structure. Schematic diagram depicting the domain organisation of 414 amino acid long human TDP‐43. TDP‐43 mainly consists of an N‐terminal domain with a nuclear localisation signal (NLS: 82–98), two RNA‐recognition motifs (RRM1: 104–176 and RRM2: 192–262), a nuclear export signal (NES: 239–250) and a C‐terminal region encompassing a hydrophobic patch (HP: 318–343) and glutamine/asparagine‐rich (Q/N:345–366) and glycine‐rich (366–414) regions. Additionally, mitochondrial localisation motifs (M1: 35–41; M3:146–150; M5: 294–300) are also shown. For comparison, a schematic of TDP‐1 (*C. elegans* TDP‐43 orthologue) is shown. TDP‐1 shows a similar structural architecture in the N‐terminus as human TDP‐43 but lacks the HP and Q/N regions in the C‐terminus. Schematic adapted and modified from Lagier‐Tourenne et al. (2010).

Structurally, TDP‐43 mainly consists of an N‐terminal domain (NTD, aa 1–102), a nuclear localisation signal (NLS, aa 82–98), two tandem RNA recognition motifs (RRM1, aa 104–176 and RRM2, aa 192–262), a nuclear export signal (NES, aa 239–250) followed by a C‐terminal glycine‐rich region (aa 274–414) (Fig. 1) (for a review, see [80]). This multidomain architecture allows TDP‐43 to engage in numerous aspects of RNA metabolism, including transcriptional regulation, RNA splicing, RNA transport, RNA stability and translation [81, 82], and many others, such as its recently reported direct involvement in the cryptic splicing event of *UNC13A* [83], a potent genetic risk factor for ALS and FTD [84, 85]. The consequences of this newly discovered function is that an increase in cryptic exon inclusion of *UNC13A* mRNA due to a mere nuclear depletion of TDP‐43 results in reduced UNC13A protein levels [83], showcasing an example of a likely genetic interaction in the context of the disease.

The N‐terminal domain resembles a disheveled and axin (DIX) domain that ensures proper folding and mediates the reversible oligomerisation of TDP‐43, which is crucial for its physiological functions, such as splicing [86, 87, 88, 89]. This is unlike the pathological TDP‐43 aggregates that are mostly irreversible and contain post‐translationally modified TDP‐43 [12, 43]. The DIX‐aided reversible self‐association also contributes to the liquid–liquid phase separation [86, 89], a crucial mechanism for cytoplasmic stress granule (SG) formation [90].

The nuclear localisation of TDP‐43 is critical to its physiology and is achieved through binding to importin‐α via its NLS located in the downstream of N‐terminal domain [91, 92, 93, 94, 95]. NLS harbours pathogenic mutations that lead to cytosolic mislocalisation of TDP‐43 and are associated with fALS [96]. Regarding TDP‐43 export from the nucleus, conflicting reports exist. Earlier studies suggested a role for the NES located in the RRM2 through its binding to exportin XPO1 [97, 98], recent studies however point to a mechanism based on passive diffusion, requiring neither NES nor XPO1, but determined by the size and transcription levels [92, 93, 94, 95, 99].

The two RRMs in TDP‐43, separated by a linker sequence, mediate RNA/DNA recognition and binding, besides engaging in protein–protein interactions, all crucial to its physiological functions in relation to RNA metabolism. Preferably, nucleic acid molecules rich in short UG/TG sequences are recognised by RRMs [100, 101], with RRM1 being indispensable and sufficient for RNA/DNA binding [6, 102]. RRM2, on the other hand, might also aid in the NTD‐mediated homodimerisation of TDP‐43 due to the presence of extra β‐strands unique to RRMs in TDP‐43 [103]. Despite the independent domain‐specific functions, nucleic acid recognition requires the interaction of both the RRMs [81]. The involvement of RRMs in RNA binding serves additional roles that are critical to the pathophysiology of TDP‐43. A recent study has revealed that expanded CAG repeats amplify the methylation of RNA to N^1^‐methyladenosine (m^1^A), facilitating and enhancing a direct binding of these methylated RNAs with RRMs in TDP‐43. This augmented interaction in turn contributes to the mis‐localisation of TDP‐43 and the formation of gel‐like aggregates, mirroring observations seen in numerous neurological diseases associated with this protein [104].

C‐terminal domain (CTD) is intrinsically disordered and consists of a segment rich in uncharged polar amino acids glutamine and asparagine (Q/N) followed by a glycine‐rich region, giving it a characteristic of prion‐like domains of yeast prion proteins [105, 106, 107]. CTD determines the solubility and folding of TDP‐43, besides regulating its cellular localisation and protein–protein interactions [74, 108, 109]. CTD is required for the splicing activities of TDP‐43 [110, 111] and self‐regulation of its own mRNA levels [112] as well as of other disease‐associated proteins for example tau [113], a protein implicated in multiple neurodegenerative diseases referred to as tauopathies (for a review please see [114]).

Under stress conditions, CTD contributes to the initiation of dynamic membraneless organelles through liquid–liquid phase separation (LLPS), a phenomenon of adaptive physiological significance [115, 116]. These droplets can however transform into irreversible pathological aggregates through liquid‐to‐solid phase separation as they age, by mutations or when the stress persists [89, 117, 118, 119, 120, 121]. In relevance to self‐association, be it the reversible LLPS or the irreversible pathological aggregation seen in TDP‐43 proteinopathies, small amino acid sequences in the otherwise well‐structured RRMs have been identified to have a propensity to misfold, and thereby contribute to phase separation by promoting the seeding and/or propagation during the pathogenic conversion of the CTD [122, 123].

Additionally, proteins that are known to interact with TDP‐43 physiologically or pathologically, were reported to modulate the phase separation of TDP‐43 CTD, with granulin‐5 for example favouring the LLPS [124], while granulin‐3 promoted the amyloid fibril formation [124]. Another interaction partner α‐synuclein might facilitate the liquid to amyloid transition of TDP‐43 CTD‐RNA liquid droplets [125]. Conversely, ubiquitin‐specific protease 10 (USP10) clears TDP‐positive stress granules preventing thereby the aberrant TDP‐43 aggregation in a mechanism that requires the RNA‐binding activity of TDP‐43 via RRMs and is independent of the deubiquitinase activity of USP10 [126]. CTD is also pathologically highly relevant since most of the disease‐linked TDP‐43 mutations [127] and phosphorylation sites are clustered in CTD, and also the catalytic C‐terminal fragments of approx. sizes 25–35 kDa make up the bulk of the inclusion bodies found in ALS‐affected brains [128, 129].

Additional structural elements in TDP‐43 include the three mitochondrial localisation signals M1, M3 and M5 that were reported to be partially responsible for its import to the mitochondria via a mechanism likely involving the TOM70/TIM22 complex [75], as well as caspase‐cleavage sites [130]. For fine structural details of various TDP‐43 domains, regions, and motifs, we refer our readers to a review by François‐Moutal *et al*. [131].

### Structural features of TDP‐1, the *C. elegans* orthologue of human TDP‐43

Encoded by *tdp‐1* gene (TAR DNA‐binding protein homologue), TDP‐1 is the only *C. elegans* protein homologous to multifunctional human TDP‐43. TDP‐1 shares significant sequence homology with TDP‐43 predominantly in the N‐terminus. Like TDP‐43, the N‐terminus of TDP‐1 contains elements that display high homology to putative RNA recognition motifs (RRM), RRM1 and RRM2, that are well known to regulate specific RNA and DNA binding. Likewise, the nuclear localisation signal (NLS) and the caspase cleavage sites present in human TDP‐43 are also present in the *C. elegans* orthologue [132]. The main distinction is that the *C. elegans* TDP‐1 C‐terminus, which in human TDP‐43 is an unstructured Gly‐ and Q/N‐rich region that plays a key role in phase separation and aggregation [133, 134], as well as exon skipping [1], is shorter and lacks the glycine rich sequence [1, 110]. At the gene level, *C. elegans* TDP‐1 contains six introns against four in the human *TARDBP* gene, and human *TARDBP* gene is thought to have lost the two introns over the course of evolution from the ancestral *TARDBP* gene [1]. In higher animals, including humans, multiple isoforms exist because of a complex alternative splicing of the primary TDP transcript, indicating highly diverse functions of the *TARDBP* gene. In contrast, only two splicing TDP‐1 isoforms are known to exist in *C. elegans*, with the longest one containing three extra amino acid residues SLQ [110]. Interestingly, all the shorter human TDP‐43 isoforms lack the Gly‐rich region [1], and like *C. elegans* TDP‐1 (which also lacks the Gly‐rich region in the C‐terminus), the shorter isoforms are unable to affect splicing regulation [110].

Since the proteins are primarily ribonucleoproteins, their RNA binding specificity matches very well. Not only does *C. elegans* TDP‐1 specifically bind UG repeats like human TDP‐43, but it also competes with human TDP‐43 for its association with specific RNAs [110] in a stoichiometric fashion. Both *C. elegans* TDP‐1 isoforms share similarities with human TDP‐43 in several other important aspects. *C. elegans* TDP‐1 proteins display a pattern of sequence specificity in recognising UG/TG‐containing RNA and DNA oligonucleotides [110], comparable to that described for human TDP‐43 [6]. Similarly, proteins from both the species require a minimum of four UG repeats to interact with oligonucleotides, can strongly bind (TG)_12_‐DNA and show no interaction with double stranded DNA. UG or TG repeats are indispensable for this interaction since protein counterparts from both the species can hardly bind RNA and DNA sequences (including the TAR DNA sequence), devoid of UG or TG repeats, or non‐TG RNA/DNA dinucleotide repeats, while the C‐terminal domain following the RRM2 is not required for interaction with RNA or DNA [110].

Another example of functional conservation between TDP‐1 and TDP‐43 is the physiological importance of salt‐bridge between the conserved RRM domains. Homologous mutations in TDP‐1, that in TDP‐43 disrupt the RRM1‐RRM2 salt‐bridge resulting in its subcellular mislocalisation and destabilisation, interference with RNA binding and substrate recognition making it consequently less neurotoxic, closely mimics the phenotype observed in *C. elegans tdp‐1* deletion mutants [135].

### Functional conservation between TDP‐43 and TDP‐1

*Caenorhabditis elegans* TDP‐1, like its mammalian homologue TDP‐43, is primarily a DNA/RNA binding protein. Under normal conditions, TDP‐1 is mainly localised to the nucleus [136], where it participates in multiple biological processes. TDP‐1 expression appears to be developmentally regulated as evidenced by higher transcript levels in oocytes and the first larval stages [137]. The expression pattern as derived from the reporter strains shows a rich expression in multiple tissues including body wall muscles, intestine, pharynx, and neurons, both in larvae and adults [136, 138]; in unstressed animals, the expression is low and primarily nuclear [136].

Although *C. elegans tdp‐1* mutants (with a 1.2 kb N‐terminal region, including the two RRM domains and the nuclear export signal deleted) are viable, the mutant worms display developmental, fecundity and locomotion defects [138]. This is in stark contrast to the higher more complex organisms where TDP‐43 homologues are necessary for survival [139, 140, 141, 142]. Interestingly, some of the defects in *C. elegans tdp‐1* mutants can be largely restored by heterologous expression of the human TDP‐43 [138], indicating a functional conservation. Conversely, this functional redundancy can also be demonstrated in transgenic worms overexpressing human TDP‐43, where the toxicity due to human TDP‐43 overexpression is attenuated by removing the endogenous worm TDP‐1 [138].

Loss of *tdp‐1* protects against the toxicity induced by aggregation‐prone 25‐KDa carboxyl fragment TDP‐C25 [138], a unique component of the TDP‐43 positive inclusions in amyotrophic lateral sclerosis (ALS) and frontotemporal lobar degeneration (FTLD) patient brain samples [11, 143]. Likewise, toxicity induced by aggregation‐prone familial ALS‐associated Cu‐Zn superoxide dismutase (*SOD‐1*) mutations [144] are ameliorated by loss of *tdp‐1* [138]. Further support for TDP‐1's role in protein quality control is apparent from experiments where the compromised growth and physiology of animals carrying a loss‐of‐function mutation in the *C. elegans* heat shock factor 1 (*hsf‐1*) [145] were improved by loss of *tdp‐1*, and this protective effect of *tdp‐1* loss appears to be specific to *hsf‐1* but not the other temperature‐sensitive mutants [138]. Further functional studies to elucidate the genetic interaction between *tdp‐1* and *hsf‐1* in proteostasis established that *tdp‐1* is acting either downstream of *hsf‐1* or in a parallel pathway [138].

Besides the above‐mentioned functions, TDP‐1 is involved in ageing pathways. *tdp‐1* mutants live longer than wild‐type worms and this negative regulation of ageing by TDP‐1 is independent of heat shock factor HSF‐1 but requires intact transcription factor DAF‐16 [138]. TDP‐1 also participates in the classical insulin/IGF‐signaling (IIS) stress‐response and longevity pathway that operates via a phosphorylation cascade through the DAF‐2/insulin‐IGF receptor. When downregulated, DAF‐2 promotes nuclear localisation of the downstream forkhead transcription factor DAF‐16 by relieving its phosphorylation, thereby promoting longevity and stress resistance via the upregulation of DAF‐16 transcriptional activity [146, 147, 148, 149, 150]. When combined with DAF‐2 deficiency, TDP‐1 loss limits the extended lifespan of *daf‐2* mutants, despite the *tdp‐1* deficient worms themselves having a longer life‐span than wild‐type worms, as well as reducing the oxidative stress resistance in DAF‐2‐deficient worms, partially by reduced expression of DAF‐16 target genes, such as *sod‐3* [136]. Conversely, stress was found to induce endogenous TDP‐1 expression [136]. Interestingly, all the above‐mentioned phenotypic changes due to *tdp‐1* loss matched with the dysregulated biological processes obtained from transcriptional and gene ontological studies in *tdp‐1* mutants [138].

Several other novel functions have been revealed and assigned to an already growing list of known TDP‐1 functions, such as its recently reported role in maintaining transcriptome and double‐stranded RNA levels [151, 152], also shared by its mammalian homologue TDP‐43, and RNA interference (RNAi) and related chromatin changes, some aspects of which are mediated through its interaction with HPL‐2, the *C. elegans* heterochromatin protein 1 homologue [153]. Other areas where the human TDP‐43 and its *C. elegans* orthologue TDP‐1 show functional conservation, is their involvement in the DNA damage response (DDR) mechanism as reported recently [154]. Analogous to TDP‐43 depletion in cellular set‐ups, TDP‐1 loss in *C. elegans* results in increased accumulation of DNA damage, more lethality and impaired repair of double‐strand breaks induced by radiation or chemicals [154]. Likewise, evidence of conservation of additional canonical functions of TDP‐43, e.g. miRNA biogenesis involving both nuclear Drosha and cytosolic Dicer complexes [155], have recently been reported in *C. elegans* TDP‐1 [156]. TDP‐1 loss decreased the overall differential miRNAs and PIWI‐interacting RNAs (piRNAs) profile observed in mutant human α‐synuclein transgenic *C. elegans* [156]. Similarly, other recently identified functions of human TDP‐43, such as retrotransposon suppression [157] and alternative polyadenylation (APA) [158], will serve as future areas of study for functional conservation due to their impact, for instance, on the expression of disease‐relevant genes, such as *ELP1*, *NEFL*, and *TMEM106B* [158].

Despite the many similarities discussed above, the two proteins behave differently *in vitro* owing to the absence of the so‐called prion‐like domain in *C. elegans* TDP‐1 C‐terminus, rendering the recombinant *C. elegans* TDP‐1 inherently incapable of forming ThT‐positive aggregates. However, upon insertion of a hydrophobic patch (residues 318–343) and Q/N sequences from human TDP‐43, modified *C. elegans* TDP‐1 chimer then aggregates in a fashion akin to human TDP‐43 [159]. Likewise, a previous study reported a major distinction between human TDP‐43 and the *C. elegans* homologue TDP‐1 in controlling the exon splicing in the context of *in vitro* functional assays, with the C‐terminal glycine‐rich region following RRMs in human TDP‐43 (which in *C. elegans* TDP‐1 is shorter and lacks the glycine rich sequence) being responsible for recognising and interacting with the splicing regulatory complex [110]. This was, however, refuted by an unrelated study where TDP‐1 supported the Cystic Fibrosis Transmembrane Conductance Regulator (CFTR) splicing in a cell‐based assay like human TDP‐43 [132]. Despite being shorter and devoid of glycine rich sequences, TDP‐1 C‐terminal domain (residues 347–411) when fused to an otherwise splicing incompetent human TDP‐43 N‐terminal domain (residues 1–269) restored the splicing ability in the resulting chimeric TDP‐43::TDP‐1 construct [132]. Further proof of these findings is provided by the recent genetic interaction studies where *C. elegans tdp‐1* mutants specifically show exon inclusion in *pqn‐41* gene that encodes a polyglutamine‐containing protein, which otherwise in wild‐type animals is skipped [160].

### Human TDP‐43 variants and the functional consequences (*in vitro* studies)

TDP‐43 encoding *TARDBP* gene in humans is located on chromosome 1 (1p36.22) and comprises six transcribed exons. Translation of exons 2–6 results in the major protein form with 414 amino acid residues. Of these exons, exon 6 alone encodes more than 70% of the entire mRNA transcript and approx. 60% of the TDP‐43 protein, including the glycine‐rich domain where a majority of the mutations (including the pathogenic mutations) occur. Since the identification of TDP‐43 as the main component of ubiquitinated protein aggregates in amyotrophic lateral sclerosis (ALS) and frontotemporal lobar degeneration (FTLD), more than 40 TDP‐43 mutations have been identified in ALS and FTLD patients immunoreactive to TDP‐43 pathology (reviewed in [24, 161, 162].

A number of these missense pathogenic mutations result in the addition or removal of residues that are potential targets of post‐translational modifications and are thereby assumed to change the biochemical characteristics of TDP‐43. For instance, mutations that create phosphorylatable residues, e.g. N267S, G287S, G295S, G298S, A315T, N352S, R361S, A382T, N390S [24, 163, 164, 165], remove phosphorylatable residues, e.g. S379C, S379P and S393L leading to for instance loss of casein kinase I directed phosphorylation [165, 166] or create ubiquitable residues, e.g. Q331K, N345K [22, 167]. Likewise, other mutations can add residues that increase the tendency for proteolysis and the consequent aggregation, e.g. D169G, A315T, M337V, N345K, I383V [24, 167, 168, 169]; render them resistant to caspase‐3 mediated digestion, e.g. A90V [170]; increase the aggregation propensity via disulphide bridge formation, e.g. G348C, S379C [164]; increase its mitochondria import, e.g. 298S, A315T or A382T [75]; increase the protein stability and its interaction with other ALS‐linked proteins, or decrease the aggregation, e.g. G298S, Q331K, and M337V, D169G and K263E [171, 172, 173]; disrupt phase separation, e.g. A321G, Q331K and M337V [121], or alter its sub‐cellular localisation, e.g. G294V, A315T, M337V, S375G, A382T, and G376D [96, 174, 175, 176]. Some of these pathogenic mutant variants have been thoroughly studied for their aggregation propensities (see Table 1).

**Table 1:** Aggregation properties of recombinant or synthetic human TDP‐43 and *C. elegans* TDP‐1 constructs assessed *in vitro* using turbidity or Thioflavin T (ThT) binding assays. The domain structure of human TDP‐43 is shown along with the C‐terminal amino acid sequence, highlighting the hydrophobic patch (HP, yellow) and glutamine‐ and asparagine‐rich region (QN, cyan). Pathogenic mutations analysed for their role in promoting TDP‐43 aggregation are indicated in red, while phosphorylation sites (pS409/410) are marked in brown. Mutant protein/peptide variants are compared for their aggregation competence, relative to the respective wild‐type (WT) protein/peptide. Yes, forms aggregates; No, does not aggregate; No change, aggregation similar to WT; MetO, methionine sulfoxidation induced by H₂O₂; phospho‐TDP, TDP peptide phosphorylated using casein kinase‐1 (CK1δ); TDP‐Ins, chimeric construct with HP and Q/N regions from human TDP‐43 inserted into *C. elegans* TDP‐1.

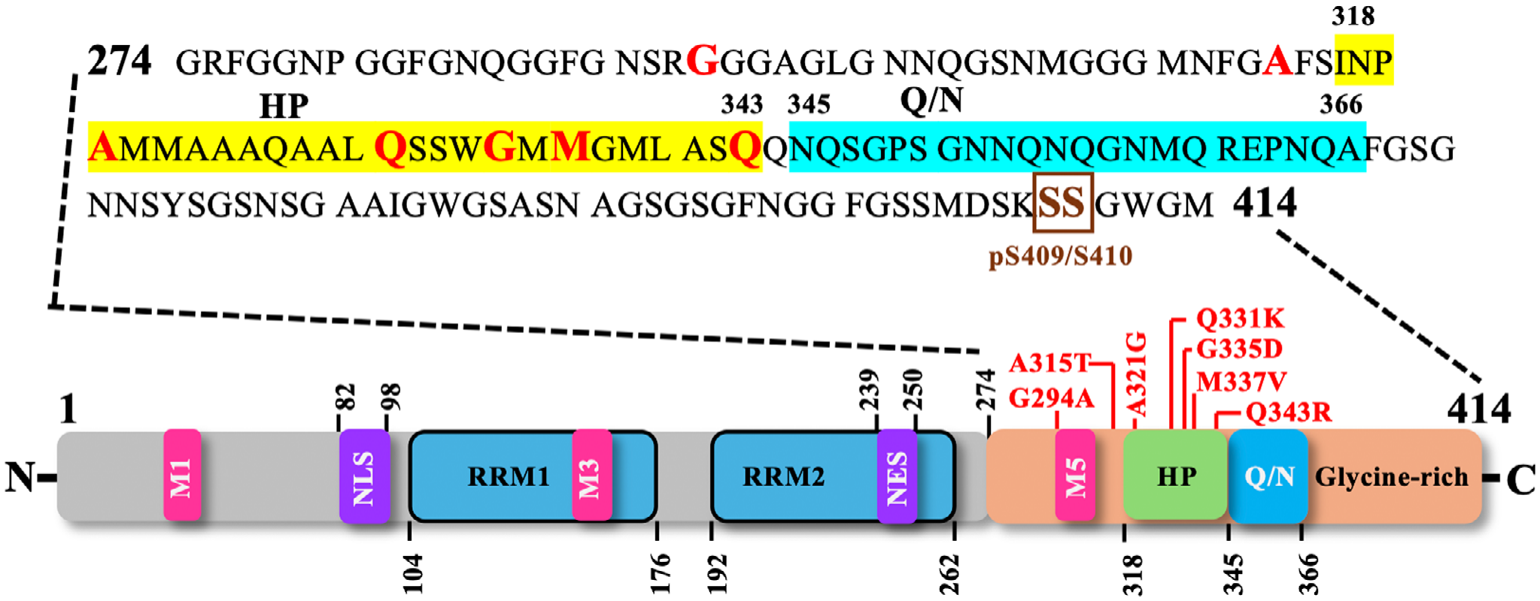		
Construct	Aggregation propensity (*in vitro*)	References
TDP43 WT	Yes	[109]
TDP‐43 G294A	No change	
TDP‐43 Q331K	Increased	
TDP‐43 M337V	Increased	
TDP (1–275)	Yes	
TDP (188–414)	No	
synthetic TDP‐43 WT peptide (Gln286‐Gln331)	Yes	[179]
synthetic TDP‐43 A315T peptide (Gln286‐Gln331)	Increased	
TDP‐43	Yes	[159]
TDP‐43 minus HP (residues 318–343 deleted)	Reduced	
TDP‐43 minus QN (residues 344–360 deleted)	Reduced	
TDP‐43 A324E/M337E	Reduced	
TDP (311–360)	Yes	
TDP (361–414)	No	
*C. elegans* TDP‐1 WT	No	
TDP‐Ins	Yes	
TDP‐43 WT	Yes	[181]
TDP (311–360) WT	Yes	[180]
TDP (311–360) G335D	Increased	
TDP (311–360) Q331K	No change	
TDP (311–360) M337V	No change	
TDP (311–360) Q343R	Reduced	
TDP (267–414) WT	Yes	[121]
TDP (267–414) A321V	Increased	
TDP (267–414) A321G	Reduced	
TDP (267–414) Q331K	Reduced	
TDP (267–414) M337V	Reduced	
TDP (267–414) A326P	Reduced	
TDP (267–414) M337P	Reduced	
TDP (267–414) minus (residues 321–343)	Reduced	
TDP (274–414)	Yes	[186]
Phospho‐TDP (274–414)	Yes	
TDP (274–414)‐MetO	Reduced	
Phospho‐(274–414)‐MetO	Reduced	
TDP‐43 WT	Yes	[182]
TDP (279–360)	Yes	

In this section, we will briefly explore the biochemical consequences of the pathogenic TDP‐43 mutant variants that have been modeled in *C. elegans*, and the consequences that have been inferred solely from *in vitro* investigations utilising purified recombinant proteins expressed in bacteria. A comprehensive review of the pathophysiological studies of mutant TDP‐43 variants, and the prevalence of the most common pathogenic mutations in sporadic/familial ALS and FTLD patients and their phenotypes have been covered elsewhere [82, 161, 177, 178].

*In vitro* studies have established that TDP‐43 is inherently prone to aggregation. For example, when incubated at 25°C with agitation, purified recombinant full‐length TDP‐43 undergoes rapid aggregation. This aggregation process requires the C‐terminal part since a fragment that lacks the C‐terminus (residues 1–275, comprising the N‐terminal domains, RRM1 and RRM2) remains soluble, also the C‐terminal fragment (residues 188–414, comprising RRM2 and the C‐terminal domain) alone is self‐sufficient to undergo aggregation with almost similar kinetics as the full‐length TDP‐43 [109]. Thus, the C‐terminal part of TDP‐43 must contain sequences with a tendency to form β‐sheets that drive its aggregation. Indeed, a 46‐amino acid sequence in the C‐terminus (residues 286–331) was identified with its N‐terminal half tending to form β‐turns and the C‐terminal half (especially residues 313–321) adopting a β‐conformation, the probability of which is increased by pathogenic mutations, such as A315T [179]. This A315T‐induced increase in β‐propensity is seemingly due to the addition of a new phosphorylatable residue, since a phosphomimetic mutant variant of the 46‐amino acid fragment (A315E) showed an even higher β‐propensity than A315T [179]. In line with this study, an amyloidogenic core (residues 311–360) containing a hydrophobic patch (residues 318–343) and a Gln/Asn‐rich motif (QN; residues 344–360) was identified to be the main driving force behind TDP‐43 aggregation [159]. As such, a recombinant amyloidogenic core peptide (residues 311–360) readily aggregates while any disruption in the amyloidogenic core (e.g. mutations in the hydrophobic patch, such as A324E/M337E, deletion of the hydrophobic patch or Gln/Asn‐rich motif) reduces the aggregation of recombinant TDP‐43 [159]. Similarly, G335D mutation increased and Q343R mutation reduced, whereas Q331K and M337V mutations did not change the aggregation propensity of the amyloidogenic core peptide (residues 311–360) [180]. Regarding full‐length TDP‐43, recombinant variants harbouring Q331K or M337V mutation showed enhanced aggregation while recombinant TDP‐43 with G294A mutation showed similar aggregation kinetics as the wild‐type, as judged by the turbidity and sedimentation assays [109].

As far as the amyloidogenic nature of TDP‐43 aggregates is concerned (as judged by their ability to bind amyloid‐diagnostic dyes Congo Red and Thioflavin T or anti‐amyloid oligomer‐specific antibody), contrasting studies have been reported, where aggregates formed by the recombinant full‐length TDP‐43‐bound ThT and the aggregation kinetics followed a typical sigmoidal curve [159], the aggregates shared epitopes with amyloid oligomers besides promoting the amyloid‐β oligomerisation [181], the aggregates bound Congo Red and Thioflavin T very weakly [182] or did not bind at all [109]. Nonetheless, aggregates formed by synthetic or recombinant truncated C‐terminal fragments containing the hydrophobic amino acids as well as those formed by RRMs are fibrillar in nature, bind ThT and follow a typical sigmoidal curve [109, 159, 179, 180, 182, 183, 184, 185, 186], similar to the prion domain of the yeast prion protein Sup35 [187].

That being said, there are some important points that need to be emphasised here. One, TDP‐43 fibres extracted from patient brains do not resemble the *in vitro* synthesised fibrils of the recombinant TDP‐43, with the filament core of the former adopting a previously unknown double‐spiral‐shaped fold, which contrasts with the folds, individual amino acid interactions, and secondary structure observed in recombinant fibrils [188]. Two, a recent study by Eisenberg and colleagues did confirm the association of TDP‐43 with the amyloid fibrils isolated from FTLD‐TDP subtype patients’ brains. However, contrary to previous beliefs, the amyloid fibrils themselves turned out not to be composed of TDP‐43 but of a 135‐residue carboxy‐terminal fragment of transmembrane protein 106B (TMEM106B) [189], previously identified as a genetic risk factor in a genome‐wide association study of 515 individuals with confirmed FTLD‐TDP [190]. Since then, an International FTLD‐TDP whole‐genome sequencing consortium has been established, and the first genome‐wide association studies from this consortium identified novel and known genetic risk factors [191]. These discoveries open up a range of questions and avenues for further research, and understanding for instance the exact role of TMEM106B fibrils in disease pathology could have significant implications for the development of therapeutic interventions.

### Transgenic *C. elegans* TDP models

In this section, we will describe *C. elegans* TDP models that have been generated to understand the pathophysiology of TDP‐43 in FTLD/ALS. Most of these models are based on the heterologous expression of human TDP‐43 using *C. elegans* neuron specific promoters (see Table 2 for promoters and their biology) with only a few exceptions where the worm TDP‐1 orthologue has been used (Fig. 1), and the organ of choice has been the nervous system, for the obvious reasons that the diseases are neurodegenerative in nature.

**Table 2:** Summary of promoters used to drive human TDP‐43 transgene expression and their biology.

Promoter sequence	Expression pattern	Confirmation method	Gene description
*egl‐3*	Pan‐neuronal	Immunostaining (egl‐3 antibody) [314]	Orthologue of human proprotein convertase subtilisin/kexin type 2 (PCSK2), involved in multiple processes including chemical synaptic transmission and protein‐processing [315]
*snb‐1*	Pan‐neuronal	*Psnb‐1::lacZ* reporter [316]	Encodes protein identical to human vesicle‐associated membrane protein 2 (VAMP2)/synaptobrevin, functions in synaptic transmission, essential for partial neurotransmitter release and *C. elegans* viability [316]
*tdp‐1*	Body wall musculature; intestine; muscle cell; neurons; and pharynx	*Ptdp‐1::yfp* reporter [138]	Encodes TAR DNA binding protein 1, identical to human transactive response DNA binding protein 43 (TARDBP) [138]
*unc‐47*	GABAergic motor neurons	*Punc‐47::gfp* reporter [309, 317]	Identical to human solute carrier family 32 member 1 (SLC32A1), involved in inhibitory neurotransmitters glycine and gamma‐aminobutyric acid transport and loading into synaptic vesicles [317, 318]
*myo‐3*	Body wall muscles	Pmyo‐3::lacZ reporter [319]	Encodes myosin heavy chain A (mhcA) that plays a role in initiating filament assembly, enabling cytoskeletal motor activity. Expressed in body wall musculature; enteric muscle; gonad; vulval muscle; and in male [320, 321, 322]

Link and colleagues [132] generated a *C. elegans* model using the synaptobrevin gene (*snb‐1*) promoter to drive the expression of human wild‐type full‐length TDP‐43 pan‐neuronally. Worms developed uncoordinated phenotypes in the larval stages that persisted throughout adulthood. Although an overt motor neuronal loss was not evident, transgenic worms displayed changes in the number and distribution of motor neuron synapses. Likewise, pan‐neuronal overexpression of worm TDP‐1 cDNA exogenously (in the presence of endogenous *tdp‐1* but absence of human TDP‐43) also resulted in uncoordinated phenotype [132]. The authors went on to explore the TDP‐43 domains that are indispensable for toxicity. GFP‐fusion constructs lacking RRM1 (residues 106–175), RRM2 (residues 193–257) or a C‐terminal region (residues 257–414) failed to produce any uncoordinated phenotype, as well as a TDP‐43 construct with an inactivated nuclear localisation signal (that restricts its localisation strictly to the cytosol), in contrast to the full‐length or other fusion TDP‐43 deletion constructs (Table 3 enlists the TDP‐43 construct variants used to study TDP‐43 proteinopathies in *C. elegans*). Interestingly, the C‐terminal TDP‐1 region (residues 347–411), that rescues the CFTR alternative splicing of N‐terminal TDP‐43 fragment (residues 1–270), also restores its normal nuclear localisation, and renders it neurotoxic in *C. elegans* [132].

**Table 3:** Human TDP‐43 and *C. elegans* TDP‐1 constructs used to model proteinopathies in *C. elegans*. Human TDP‐43 domain structure depicting the pathological mutations (in red) that have been modeled in *C. elegans*. HP: Hydrophobic Patch (yellow); Q/N: Glutamine‐ and Asparagine‐rich region (cyan); pathological phosphorylation sites (brown boxed); TDP‐43^N‐terminus (residues 1–270)^ + TDP‐1^C‐terminus (residues 347–411)^: a chimera with N‐terminal human TDP‐43 fused to C‐terminal *C. elegans* TDP‐1; blank spaces labeled by (−): not reported/investigated.

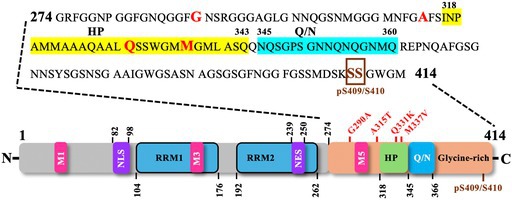				
TDP‐43 constructs	Localisation	Detergent‐insoluble aggregates	Toxicity	References
TDP‐43	Nuclear	(−)	Yes	[132]
GFP‐TDP‐43	Nuclear	(−)	Yes	
GFP‐TDP‐43 ΔRRM1 (residues 106–175)	Nuclear	(−)	No	
GFP‐TDP‐43 ΔRRM2 (residues 193–257)	Nuclear	(−)	No	
GFP‐TDP‐43 ΔC‐terminus (residues 257–414)	Nuclear	(−)	No	
TDP‐43 no NLS (K82S, R83S K84S)	Cytoplasmic	(−)	No	
TDP‐43 no caspase (D89E, D219E)	Nuclear	(−)	Yes	
TDP‐43^N‐terminus (residues 1–270)^ + TDP‐1^C‐terminus (residues 347–411)^ chimer	Nuclear	(−)	Yes	
GFP‐TDP‐1	Nuclear	(−)	Yes	
TDP‐43	Nuclear	Yes	Yes	[192]
TDP‐43 (G290A)				
TDP‐43 (A315T)				
TDP‐43 (M337V)				
TDP‐43 (S409E/S410E) TDP‐43 (S409D/S410D) TDP‐43 (S409D) TDP‐43 (G290A) + (S409A/S410A) TDP‐43 (M337V) + (S409A/S410A)	(−) (−) (−) (−) (−)	(−) (−) (−) (−) (−)	≈ WT ≈ WT ≈ WT Reduced Reduced	
TDP‐43‐YFP TDP‐43 (Q331K)‐YFP TDP‐43 (M337V)‐YFP TDP‐C25_(219–414)_‐YFP	Nuclear Nuclear Nuclear Cytoplasmic	Yes (−) (−) Yes	Yes Yes Yes Reduced	[143]
TDP‐43 TDP‐43 (A315T)	(−)	Yes	Yes	[206]
TDP‐43	(−)	(−)	Yes	[209]
TDP‐1 (R219A knock‐in)	(−)	(−)	≈ TDP‐1 loss	[135]
GFP control	(−)	(−)	No	[204]
TDP‐GFP‐C220_(220–414)_	(−)	(−)	Yes	
TDP‐GFP‐C233_(233–414)_	(−)	(−)	Yes	
Humanised models (endogenous TDP‐1 replaced by TDP‐43)				
TDP‐43 WT	(−)	(−)	No	[223]
TDP‐43 (M337V)	(−)	(−)	No	
TDP‐43 (A315T)	(−)	(−)	No	
TDP‐43 (G295S)	(−)	(−)	Yes	
TDP‐43 (G298S)	(−)	(−)	No	
TDP‐43 (G294A)	(−)	(−)	No	

In a different study by Kraemer and colleagues [192], transgenic *C. elegans* lines were generated to compare the pathology induced by wild type TDP‐43 against three common pathogenic mutant variants implicated in familial ALS in relation to G290A [193], A315T [24, 194], and M337V [22, 167]. The wild‐type and the ALS‐associated TDP‐43 mutant variants in *C. elegans* neurons produced distinct toxicity. While both wild‐type and the three mutant TDP‐43 variants resulted in an uncoordinated phenotype that was exacerbated with ageing, the mutant TDP‐43 variants proved more toxic [192]. The transgenic worms accumulated detergent‐insoluble aggregates of full‐length as well as truncated and high molecular weight aggregated forms of TDP‐43 [192], a characteristic feature of ALS and FTLD‐U [11], with wild‐type TDP‐43 preferably isolating in the insoluble fraction. Despite this, mutant TDP‐43 variants but not the wild‐type over‐expression resulted in neurodegeneration manifested in the form of neuronal abnormalities and loss of cell bodies [192]. While the truncated products were seen in the TDP‐43 transgenic worms, cell death caspases did not participate in the locomotor defects, quantity of truncated species produced or the neuronal degeneration [192], consistent with an independent study whereby a mutant TDP‐43 construct was rendered caspase‐insensitive due to D89E/D219E substitutions inducing neurotoxicity like wild‐type TDP‐43 [132].

Another distinction is the post‐translational modifications of TDP‐43 (ubiquitination and pathological phosphorylation at S409/S410) characteristic of ALS and FTLD‐U [11, 12, 43] that the transgenic worms were able to recapitulate. While high molecular weight ubiquitinated protein proportionate to total TDP‐43 levels was found in all the TDP‐43 transgenic lysates, phosphorylated protein at the same time differed among the transgenic lines. Mutant TDP‐43 lysates (soluble and insoluble) in general showed more phosphorylation than the wild‐type which showed pathological phosphorylation only in the detergent‐insoluble fraction [192]. Transgenic lines with higher wild‐type TDP‐43 expression levels, however, resisted any phosphorylation. Using TDP‐43 constructs that lack the two pathological phosphorylation sites (serine residues at 409/410 mutated to alanine), the authors demonstrated that the phosphorylation is necessary for neurotoxicity induced by mutant but not the wild‐type TDP‐43 in transgenic worms [192]. While it may be argued that the differential phosphorylation of TDP‐43 in distinct transgenic lines is directly linked to the differential pathological outcome, the precise role of phosphorylation in pathology in this and multiple proteinopathies involving other proteins nonetheless remains uncertain. Using these mutant TDP‐43 *C. elegans* transgenic worms, Liachko and colleagues investigated the in vivo consequences of molecular chaperone HSP‐90/DAF‐21 inhibition [195] on TDP‐43 toxicity, building upon earlier *in vitro* work which however had contrasting findings [196, 197]. Loss of HSP‐90 protected against TDP‐43 neurotoxicity and subsequent neurodegeneration in adult worms, as was also achieved via its pharmacological inhibition by tanespimycin, and further requiring small heat shock protein HSP‐16.1 [195]. Beyond phosphorylation, ubiquitination and proteolytic processing [11, 77], TDP‐43 also undergoes SUMO2/3 modifications, which may indirectly reduce its cytoplasmic aggregation by promoting its nuclear retention [198] and influencing its splicing activity [199]. While ubiquitinated TDP‐43 fragments can be toxic if not degraded and phosphorylation affects its solubility within condensates [77, 200, 201], a recent study showed that SUMO2/3 modification preferentially in the cytoplasmic pool of RNA‐free TDP‐43 reduces its aggregation under oxidative stress [202]. Using arsenic‐induced oxidative stress in *C. elegans* overexpressing TDP‐1‐mCherry and SUP46‐GFP—a RNA binding protein recruited inside stress granules (SG) [203]—both proteins formed cytoplasmic foci, whose mobility decreased with increasing arsenite concentration [202]. Furthermore, RNAi silencing of SUMO‐conjugating enzyme Ubc9 reduced TDP1‐mCherry mobility even without stress and caused complete immobilisation under low‐dose arsenite [202]. These findings, along with human cell experiments, suggest SUMOylation protects TDP‐43 from aggregation under stress.

Of note, in the TDP‐43 transgenic worms described so far, the endogenous *C. elegans* TDP‐1 apparently did not participate in the human TDP‐43 induced neurotoxicity and the TDP‐43 inclusions were exclusively localised to the neuronal nuclei [132, 192]. However, the TDP‐43 inclusions in one case were ubiquitin‐negative [132], whereas in the other the inclusions were ubiquitin‐positive [192].

Similarly, transgenic *C. elegans* over‐expressing pan‐neuronally the wild‐type or ALS‐associated mutant TDP‐43 variants (Q331K and M337V) tagged with yellow fluorescent protein (YFP) at C‐terminus show neurotoxicity manifested by locomotor defects, defective synaptic transmission, slow growth and accumulation of detergent‐insoluble aggregates in the absence of frank neurodegeneration, with mutant YFP‐TDP‐43 variants inducing more toxicity than the wild‐type YFP‐TDP‐43 [143], similar to the untagged TDP‐43 variants described earlier [132, 192]. The same group also used the *C. elegans* endogenous *tdp‐1* promoter to drive the overexpression of full‐length human wild‐type TDP‐43 and reported similar locomotor defects and temperature sensitive developmental defects [138]. Additionally, transgenic *C. elegans* over‐expressing YFP‐tagged TDP‐C25 (residues 219–414), a truncated aggregation‐prone C‐terminal TDP‐43 fragment found in ubiquitinated inclusions in brain and spinal cords of FTLD and ALS patients [11], had moderate toxicity than the full‐length YFP‐tagged TDP‐43 over‐expressing worms despite accumulating more insoluble or less diffusible aggregates [143]. Although all the YFP‐tagged TDP‐43 transgenic lines in general showed temperature sensitivity, worms overexpressing the YFP‐TDP‐C25 fragment were more vulnerable to high temperatures [143]. Additionally, suppressing the insulin/IGF‐signaling (IIS) ameliorated the toxicity in the YFP‐tagged TDP‐43 transgenic worms [143]. Likewise, depletion of endogenous *C. elegans* TDP‐1 [138] protected against toxicity induced by YFP‐tagged TDP‐C25 in transgenic worms, contrary to the earlier models where endogenous TDP‐1 played no role in neurotoxicity, induced however by full‐length TDP‐43 overexpression [132, 192]. Similarly, using a range of GFP‐tagged carboxy‐terminal fragments of human TDP‐43 in a cellular model, Kinjo and colleagues demonstrated that a part of RRM2 is sufficient to induce structural transition of the intrinsically disordered region (IDR) in the carboxy‐terminus, driving the condensation and aggregation [204]. Out of these constructs, two GFP‐tagged carboxy‐terminal fragments—GFP‐C220 (residues 220–414) and GFP‐C233 (residues 233–414)—were expressed in the body wall muscles of *C. elegans* to investigate their condensation properties and associated toxicity in transgenic worms. While both the constructs generally produced toxicity compared to GFP alone, GFP‐C220 showed a higher tendency to oligomerise, formed condensates that were less fluidic and was more toxic than GFP‐C233 [204].

A pan‐neuronal model is preferable due to the reinforced validity that it offers if a particular class of neurons shows increased vulnerability over others (for an example see [205]). Nonetheless, transgenic *C. elegans* with targeted transgene expression in a subpopulation of neurons have been created. Concerning ALS, which is a motor neuron disease, wild‐type and mutant TDP‐43 expression targeted to GABAergic motor neurons using *unc‐47* promoter were generated [206]. In this case also, mutant TDP‐43‐A315T compared to wild‐type TDP‐43 induced more severe neurotoxicity manifested by locomotor defects, motor dysfunction and degeneration of motor neurons [206]. Notably, this neurodegeneration could be mitigated by inhibiting the toll interleukin 1 receptor domain adaptor protein TIR‐1 pathway and the transcription factor ATF‐7 [207]. Similarly, compared to the wild‐type TDP‐43, most of which segregated in the soluble fraction, mutant TDP‐43A315T mostly remained insoluble, but neither of the two variants (wild‐type or mutant TDP‐43A315T) affected the lifespan [206]. Besides, the paralysis and the progressive motor neuronal demise that appeared to be largely necrotic, requiring the activities of Ca^2+^‐regulated calpain protease TRA‐3 and the aspartyl protease ASP‐4, could be rescued by reducing the intracellular Ca^2+^ levels [208]. Furthermore, the group showed that mutant TDP‐43 and FUS (but not Ataxin‐3‐Q14 and Ataxin‐3‐Q89) overexpression induces a systemic TIR/Sarm1‐dependent innate immune response that requires the neurosecretory proteins UNC‐13 and UNC‐31, and which in turn can be mitigated by suppressing the downstream kinases involved in the cascade, namely NSY‐1/MAP3K, SEK‐1/MAPK and PMK‐1/p38 MAPK [207]. In yet another model, wild‐type TDP‐43 pan‐neuronal overexpression in *C. elegans* led to locomotor defects that worsened with a heterozygous loss of progranulin [*pgrn‐1*(+/−)] but remained unaffected with its complete loss [*pgrn‐1*(−/−)]. However, no impairment of other motor functions, such as pharyngeal pumping and defecation were found, and the worms showed normal larval development and lifespan [209].

TDP‐43 autoregulates its own expression tightly under physiological conditions. In mammals, both loss and overexpression of TDP‐43 can result in neurotoxicity (reviewed in 178, 210). Notably, overexpression of wild‐type human TDP‐43 in rodent models has been shown to induce motor deficits and neurodegeneration in the absence of cytoplasmic aggregates, likely reflecting toxic gain‐of‐function via excessive nuclear activity, including aberrant splicing or repression of essential transcripts [211, 212, 213]. Also, in *C. elegans* overexpression of wild‐type human TDP‐43 is toxic, although mildly. Importantly, mutation of the RNA recognition motifs (RRMs) reduces this toxicity [132], suggesting that overactive RNA‐binding and splicing repression by TDP‐43 are indeed major contributors to the phenotype —consistent with findings in vertebrate systems. These studies reinforce that overactivity‐related toxicity is a key consideration when interpreting results from overexpression models. Nevertheless, while such models may not fully recapitulate human pathology, they remain valuable for understanding dose‐dependent thresholds, domain‐specific functions, and early cellular responses to TDP‐43 dysregulation.

Neuropathology in most human TDP‐proteinopathies shows insoluble TDP‐43 inclusions that often bear several PTMs, including phosphorylation, ubiquitination, and cleavage into toxic C‐terminal fragments, predominantly in the cytoplasm [11, 12, 214, 215]. This feature, although recapitulated in disease models like yeast and drosophila [216, 217], is less common in other models that include cell‐culture, *C. elegans* and some mice, where TDP‐43 inclusions are often nuclear [132, 143, 192, 218]. This highlights that cytoplasmic deposition is not a prerequisite for toxicity in these models and underscores the importance of species‐specific mechanisms in TDP‐43 regulation and aggregation. Nevertheless, recent studies show that most of the neurotoxic features that are associated with mutant TDP‐43 overexpression in transgenic *C. elegans*, including altered localisation, pathological phosphorylation, motor dysfunction, decreased pharyngeal pumping, disrupted chemotaxis, reduced fecundity etc. can be faithfully recapitulated in wild‐type TDP‐43 overexpressing worms by modulating the culture conditions, such as temperature [219, 220, 221]. This observation is somewhat similar to the temperature sensitivity and/or temperature‐dependent severity in neurotoxicity seen in wild‐type TDP‐43 and YFP‐tagged TDP‐43 transgenic worms reported earlier [143]. Using computational, pharmacological and genetic methods, the same group reported that TDP‐43 toxicity causes defective inhibitory GABA and excitatory acetylcholinergic transmission, with the former likely to be impaired due to fewer neuromuscular junctions (NMJs) [222]. Reduced acetylcholine release, although not utterly responsible for the paralysis in TDP‐43 worms, is contributed by upstream G‐protein‐signaling in the otherwise functional synaptic machinery of the cholinergic neurons. Consequently, neurotransmission (and only minimal locomotion) is restored by enhancing acetylcholine neuron output via G‐protein‐coupled receptors. However, rebalanced excitatory‐to‐inhibitory (E/I) ratio by synergistic stimulation of GABA and acetylcholine neurons leads to enhanced neurotransmission and a partial restoration of locomotion [222].

All the models described thus far are overexpression models generated using classical transformation methods that incorporate multiple transgene copies randomly in the *C. elegans* genome. Lately, the generation of a humanised transgenic worm harbouring a single copy of the human *TARDBP* (*tdp‐43*) transgene has been made possible by the recent advancements in genetic manipulation techniques. Using CRISPR/Cas9 genome‐editing technology, the *C. elegans tdp‐1* exons and introns were deleted and replaced by sequences encoding human TDP‐43 [223]. The CRISPR‐generated *tdp‐1* deletion primarily under stressful conditions resulted in neurodegeneration, and this neurodegeneration phenotype was rescued by human TDP‐43 replacement. Additionally, 5 FTD/ALS‐associated pathogenic mutations (M337V, A315T, G295S, G298S and G294A) were inserted in this humanised *tdp‐43* transgenic worm. While four out of these five mutations behaved like the wild‐type TDP‐43 in rescuing the neurodegeneration associated with *tdp‐1* swap, G295S mutation worsened the neurodegeneration phenotype [223]. In conclusion, the *C. elegans* nervous system serves as a valuable model to study disease mechanisms associated with TDP‐43 loss‐ and gain‐of‐function in TDP‐43 proteinopathies.

While these pioneering studies laid the groundwork for our current understanding, there are notable caveats in the interpretation of *tdp‐1* null mutant studies from the primary genetic lesions used in early research, which do not constitute certain true null mutants or might very well contain a linked background mutation. Unpublished data from CRISPR‐generated *tdp‐1* deletions [223] have not entirely replicated early findings on TDP‐1's effect on lifespan and protein‐aggregate‐driven disease‐relevant phenotypes, underscoring the need for cautious interpretation of past findings.

More importantly, TDP‐43 toxicity is now widely accepted to result from a combination of toxic gain‐of‐function effects, often related to cytoplasmic mislocalisation, aggregation, or aberrant RNA binding, and loss of nuclear function, including failure to regulate key RNA targets [210, 224, 225, 226]. These processes are typically modeled by (i) transgenic overexpression of wild‐type or mutant TDP‐43, and (ii) deletion or knockdown of the endogenous TDP‐43 homologue (*tdp‐1* in *C. elegans*), respectively, as described above.

In *C. elegans*, *tdp‐1* deletion mutants are viable and do not show overt neurodegeneration, suggesting some degree of functional divergence or redundancy [138, 151]. However, transcriptomic studies have shown that TDP‐1 regulates alternative splicing of numerous genes in *C. elegans*, indicating functional conservation of RNA binding and splicing repression as described in the earlier sections [151, 227]. At the same time, it is important to emphasise that many human TDP‐43 targets involved in disease pathology (e.g. *STMN2*, *UNC13A*) are not conserved at the level of their intron architecture or binding motifs in worms or even in rodents [225, 227]. For instance, *STMN2* cryptic exon inclusion—a hallmark of TDP‐43 loss‐of‐function in human ALS/FTD neurons—is not observed in TDP‐43 knockdown mice due to a lack of conserved intronic sequences flanking the cryptic exon [227, 228]. Thus, modeling such disease‐relevant loss‐of‐function targets is currently not feasible in *C. elegans* and highlights an inherent limitation of using invertebrate systems for certain aspects of TDP‐43‐related RNA dysregulation.

### Bigenic models

Neurodegenerative diseases are multifactorial in nature, involving several genetic, epigenetic, and environmental factors that contribute to the emergence of pathology [229, 230]. It is therefore not surprising that the presence of insoluble aggregates composed of multiple proteins present a common feature of multiple disorders. In certain instances, this correlation may result from the aggregation process itself, leading to the sequestration of other proteins nearby, while in others the pathology may emerge from genetic and functional interplay between the various factors involved. This highlights the need for further research into the interactions between different proteins in these aggregates and their contributions to disease progression. Due to its easy genetic manipulability, *C. elegans* models involving the manipulation of multiple genes simultaneously have been created to study genetic interactions and their effects on various biological processes and/or diseases. These models are instrumental in understanding complex gene networks, pathway crosstalk, and the modulation of phenotypes. Also, by studying selective combinations of disease‐implicated genes or proteins in bigenic models, researchers can identify personalised therapeutic strategies tailored to individual patients. Understanding which combinations of genes contribute most significantly to disease pathology can inform the development of targeted therapies aimed at modulating specific molecular pathways.

Concerning ALS/FTLD, the pathological lesions in these diseases consist of TDP‐43 inclusions along with UBIQUILIN‐2 (UBQLN2) [231], another protein implicated in ALS or ALS/FTLD [231, 232, 233], and in this case the two proteins are known to also interact physically *in vitro* [234]. A bigenic *C. elegans* model tested whether the wild‐type TDP‐43—that alone induces only a moderate neurotoxicity [192]—could function together to enhance the susceptibility to neurodegeneration [235] when combined with UBQLN2. Indeed, the two proteins when overexpressed together pan‐neuronally produced a severe pathology—which is apparent as severe motor dysfunction accompanied by increased neurodegeneration (see Table 4) and total expression levels of the two proteins—than worms overexpressing either of the two proteins alone [235].

**Table 4:** Bigenic TDP‐43 *C. elegans* models generated to study any genetic interaction between TDP‐43 and other disease‐associated risk factors. The consequences of a likely interaction result in ameliorated (A) or exacerbated (E) phenotype, or an unchanged (U) phenotype in the absence of any likely interaction.

Promoter sequence	Expression pattern	Transgenes involved	Phenotype ameliorated (A), exacerbated (E), or unchanged (U)?	References
htt57Q128‐CFP: *Pmec‐3*, *tdp‐1(ok803)* (TDP‐1 deleted)	Mechanosensory neurons	htt57Q128‐CFP combined with TDP‐1 loss	A	[241]
htt57Q128‐CFP: *Pmec‐3*, *fust‐1(tm4439)* (FUST‐1 deleted)	Mechanosensory neurons	htt57Q128‐CFP combined with FUST‐1 loss	A	
htt57Q128‐CFP: *Pmec‐3*, *tdp‐1(ok803)* (TDP‐1 deleted), *hdac‐6 (ok3203)* (HDAC6 deleted)	Mechanosensory neurons	htt57Q128‐CFP combined with TDP‐1 and HDAC6 losses	U	
htt57Q128‐CFP: *Pmec‐3*, *tdp‐1(ok803)* (TDP‐1 deleted), *pgrn‐1 (tm985)* (progranulin deleted)	Mechanosensory neurons	htt57Q128‐CFP combined with TDP‐1 and progranulin losses	A	
TDP‐43: *Pegl‐3*, Granulin 1, 2 or 3: *Pgrn‐1*	Pan‐neuronal	TDP‐43 WT + Granulin 1	U	[209]
	Pan‐neuronal	TDP‐43 WT + Granulin 2	E	
	Pan‐neuronal	TDP‐43 WT + Granulin 3	E	
Tau: *Paex‐3*, Granulin 3: *Pgrn‐1*	Pan‐neuronal	Tau WT + Granulin 3	U	[254]
TDP‐43: *Psnb‐1*, HASNWT: *Paex‐3*	Pan‐neuronal	TDP‐43 WT+ α‐synuclein WT	E	
TDP‐43: *Psnb‐1*, HASNA53T: *Paex‐3*	Pan‐neuronal	TDP‐43 WT+ α‐synuclein A53T	E	
HASNWT: *Paex‐3*, *tdp‐1(ok803)* (TDP‐1 deleted)	Pan‐neuronal	Humanα‐synuclein WT combined with TDP‐1 loss	A	
HASNA53T: *Paex‐3*, *tdp‐1(ok803)* (TDP‐1 deleted)	Pan‐neuronal	Humanα‐synuclein (A53T mutant) combined with TDP‐1 loss	A	
TDP‐43: *Psnb‐1*, UBIQUILIN‐2: *P*rgef‐1	Pan‐neuronal	TDP‐43 WT + UBIQUILIN‐2 (WT)	E	[235]
	Pan‐neuronal	TDP‐43 WT + UBIQUILIN‐2 (P497H)	E	
	Pan‐neuronal	TDP‐43 WT + UBIQUILIN‐2 (P506T)	E	
HRPA‐1HsLC‐D290V: *Pmec‐4*, tdp‐1(ok803) (TDP‐1 deleted)	Mechanosensory neurons	Chimeric HRPA‐1HsLC‐D290Vcombined with TDP‐1 loss	A	[255]
HRPA‐1HsLC‐D290V: *Pmec‐4*, tdp‐1(tgx58) (TDP‐1 deleted)	Mechanosensory neurons	Chimeric HRPA‐1HsLC‐D290Vcombined with TDP‐1 loss	A	
HRPA‐1HsLC‐D290V: *Pmec‐4*, FynY531F: *Posm‐10*	Mechanosensory neurons, Sensory tail neurons	Chimeric HRPA‐1HsLC‐D290V + Fyn kinase (constitutively active)	A	
TDP‐43: *Psnb‐1*, Tau: *Paex‐3*	Pan‐neuronal	TDP‐43 WT + Tau WT	E	[238]
TDP‐43: *Psnb‐1*, A‐β_1‐42_: *Punc‐119*	Pan‐neuronal	TDP‐43 WT + A‐β_1‐42_	U	
TDP‐43: *Psnb‐1*, A‐β_1‐42_: *Psnb‐1*	Pan‐neuronal	TDP‐43 WT + A‐β_1‐42_	U	
TDP‐43: *Psnb‐1*, Q86‐YFP: *PF25B5.3*	Pan‐neuronal	TDP‐43 WT + poly‐glutamine (Q86‐YFP)	U	
Dendra2::tau: *Psnb‐1*, TDP‐43: *Psnb‐1*	Pan‐neuronal	Dendra2‐tau + TDP‐43 WT	E	[239]
Dendra2: *Psnb‐1*, TDP‐43: *Psnb‐1*	Pan‐neuronal	Dendra2 + TDP‐43 WT	E	
TDP‐43: *Psnb‐1*, *pgrn‐1 (tm985)*	Pan‐neuronal	TDP‐43 (A315T mutant) combined with progranulin‐1 loss	E	[245]
TDP‐43: *Psnb‐1*, PGRN‐1: *Ppgrn‐1*	Pan‐neuronal	TDP‐43 (A315T mutant) combined with progranulin‐1 over‐expression	A	
Psnb‐1	Pan‐neuronal	Interspecies fusion construct: TDP‐43_(1‐269)_‐TDP‐1_(347–411)_	Neurotoxic	[132]

In a different study, TDP‐43 was tested to understand the susceptibility to neurotoxicity induced when combined with tau, A‐β or poly‐glutamine—proteins implicated in multiple neurodegenerative diseases [236]—and the fact that TDP‐43 co‐pathology worsens the clinical symptoms in AD [49, 50]. *C. elegans* transgenic worms were generated using a genetic manipulation technique [237] that allowed low pan‐neuronal TDP‐43 expression, and crossed with transgenic lines over‐expressing tau, A‐β or poly‐glutamine [238]. Only one combination (that of TDP‐43 and tau) resulted in exacerbated neurotoxicity, manifested by worsened motor dysfunction and mechanosensation, more total and phosphorylated tau accumulation, and selective neuronal loss [238]. In a similar fashion, TDP‐43 co‐expression exacerbated the Dendra2‐tagged tau phenotype in transgenic worms [239].

In yet another study, a genetic interaction between *C. elegans* orthologues of TDP‐43 and FUS, *tdp‐1* and *fust‐1*, respectively, with polyglutamine proteins using a transgenic *C. elegans* expressing mutant huntingtin with 128 polyQ repeats in mechanosensory neurons [240] was investigated [241]. Loss of *tdp‐1* or *fust‐1* reduced the neurotoxicity in the transgenic worm expressing the mutant huntingtin‐polyQ, as was also the case in striatal cells from huntingtin knock‐in mice after knocking down TDP‐43 or FUS, using small interfering ribonucleic acid (siRNA) [241]. While the regulation of huntingtin‐polyQ neurotoxicity by TDP‐43 required the growth factor progranulin (PGRN)–also implicated in FTLD‐U [42, 242, 243]—the same by FUS however did not [241]. In the context of TDP‐43 proteinopathy, a genetic interaction between TDP‐43 and progranulin was also studied in *C. elegans*. While a complete loss of endogenous progranulin in *C. elegans* did not affect the TDP‐43 toxicity, partial loss exacerbated the toxicity [209]. Further investigations pointed to the role of progranulin cleavage products (granulins) in TDP‐43 toxicity. When expressed exogenously, *C. elegans* progranulin cleavage products (specifically granulin 2 and 3 but not 1) exacerbated the TDP‐43 toxicity irrespective of the presence or absence of endogenous progranulin‐1 [209, 244]. This increased toxicity is likely due to increased steady‐state levels of TDP‐43 because of granulin 3‐mediated impaired degradation of TDP‐43 [244]. A more recent study investigated the contribution of progranulin‐1 to phenotypic outcomes in multiple transgenic *C. elegans* neurodegenerative models by utilising the progranulin‐1 over‐expression and *pgrn‐1* deletion scenarios. Progranulin‐1 overexpression showed a protective effect across the models tested including the mutant TDP‐43 model, whereas an aggravated neurotoxicity was reported due to loss of progranulin‐1. In selective models, however, that included the mutant TDP‐43 model [245]. Taken together, the findings from these two independent groups tell us that increased granulin‐3 levels antagonise the protective effects of progranulin‐1 in the context of TDP‐43 proteinopathies. Further support to the role of granulins in TDP‐43 proteinopathies comes from *in vitro* studies where a direct interaction between recombinant proteins of TDP‐43 C‐terminal domain and granulins were demonstrated, and individual granulins were found to differentially modulate TDP‐43 liquid–liquid phase separation and its assembly into ThT‐positive aggregates [124].

Another bigenic model investigated the contribution of chromosome 9 open reading frame 72 (*C9ORF72*), hexanucleotide repeat expansions in the intronic region of which is associated with ALS and FTD [15, 17, 246], to TDP‐43 pathology in *C. elegans*. Worms lacking a functional *C9ORF72* orthologue ALFA‐1 show age‐dependent motor dysfunction, neurodegeneration of GABAergic motor neurons and sensitivity to osmotic stress, and when combined with TDP‐43 but not FUS transgene overexpression, the motor dysfunction became worse, thereby demonstrating a differential interaction between these ALS‐ and FTD‐associated genes [247].

In addition, a transgenic worm based on a hybrid construct consisting of the N‐terminal part of human TDP‐43 fused to the C‐terminal part of *C. elegans* TDP‐1, displayed neurotoxicity similar to transgenic worms overexpressing full‐length human TDP‐43 [132]. This is not surprising, because a similar hybrid protein with hydrophobic patch (residues 318–343) and Q/N sequences (Gln/Asn‐rich motif, residues 344–360) from human TDP‐43 inserted into *C. elegans* TDP‐1 behaved like human TDP‐43 *in vitro* [159].

To understand the interaction between TDP‐43 and α‐synuclein, another protein implicated in Parkinson's disease [248], and the aggregates—both of which co‐occur in multiple neurodegenerative diseases [249, 250, 251, 252, 253]—Shen et al. used two complementary strategies [254]. In the first, endogenous *tdp‐1* in transgenic worms overexpressing wild‐type or mutant human α‐synuclein was deleted, which resulted in amelioration of α‐synuclein‐mediated neurotoxicity. And secondly, human TDP‐43 overexpression in transgenic worms overexpressing wild‐type or mutant human α‐synuclein (in the presence of endogenous TDP‐1) led to exacerbation of α‐synuclein‐mediated neurotoxicity, hence showing that the interaction between α‐synuclein and TDP‐43 is synergistic [254]. Using small RNA sequencing, the same group subsequently identified differentially regulated miRNAs and piRNAs in mutant against wild‐type human α‐synuclein transgenic worms, whose number decreased in the background of *tdp‐1* loss [156]. Some of the predicted targets of these differentially regulated miRNAs and piRNAs—especially cel‐miR‐1018, cel‐miR‐355‐5p (C34F6.1 and C05C10.3), cel‐miR‐800‐3p, and 21ur‐1581—were genes related to neuronal function, such as *T28F4.5*, *C34F6.1*, *C05C10.3*, *camt‐1*, and *F54D10.3* [156].

Similarly, an unrelated study showed that the endogenous *tdp‐1* expression is necessary to induce neurodegeneration under stressful conditions in a transgenic worm expressing a chimeric protein HRPA‐1HsLC‐D290V [255], *C. elegans* hnRNPA2 orthologue with homologous pathogenic D290V mutation, and with its low complexity (LC) domain and the third exon replaced with the corresponding human sequences of the multisystem proteinopathy (MSP)‐associated RNA‐binding protein hnRNPA2 [256], again showcasing a clear depiction of a genetic interaction between the two in the disease context. Further, this stress‐induced neurodegeneration is reduced when a constitutively active form of Fyn kinase, known to phosphorylate hnRNPA2 [257], is co‐expressed alongside HRPA‐1HsLC‐D290V in double transgenic worms [255].

Hence, the disease models described thus far exhibit a wide range of phenotypic outcomes, reflecting the diverse manifestations of neurodegenerative diseases in humans. And it is clear from the bigenic models that not all combinations of disease‐implicated proteins/genes modulate pathology. Instead, specific combinations may have synergistic or antagonistic effects on disease progression, reinforcing the complexity of neurodegenerative diseases, as well as guaranteeing comprehensive research into understanding the pathway crosstalk for deciphering the underlying mechanisms of disease and identifying potential therapeutic targets.

Considering this complexity, it is worthwhile noting that, despite the valuable insights provided by these models, the *C. elegans* system has inherent limitations when it comes to faithfully modeling human neurodegenerative diseases. For instance, it lacks a centralised brain with homologous regions, such as cortex or hippocampus, as well as the complex neural circuitry required for higher order cognitive, behavioural, and motor planning functions. This limits its use in modeling fine motor deficits, muscle denervation or electromyographic changes, or the selective vulnerability of upper and lower motor neurons seen in disorders, such as ALS. Furthermore, although *C. elegans* possesses a limited innate immune system, which recent discoveries suggest is more robust than thought earlier [258]— it lacks a vertebrate‐like adaptive immunity, including canonical glial cell types such as astrocytes and microglia, which play critical roles in neuroinflammation, synaptic support, and immune surveillance in the mammalian nervous system. Nonetheless, *C. elegans* does possess a few simple glia‐like cells, such as amphid sheath and socket cells, which can modulate stress responses and proteostasis in neurons, exerting non‐cell autonomous effects that partially resemble glial support in higher organisms [259]. Despite this, the absence of true astrocytes and microglia restricts its use for studying complex neuroimmune interactions involved in many human neurodegenerative diseases, including ALS or FTD. Additionally, the organism exhibits reduced genetic redundancy compared to humans, where gene families often contain multiple isoforms with overlapping functions. This simplification may obscure the phenotypic consequences of gene mutations or interactions.

### Genetic modulators of TDP‐43 proteinopathies

*Caenorhabditis elegans* has been instrumental in uncovering genes and pathways involved in various biological processes, including development, ageing, behaviour, and disease. *C. elegans* genome, although only 1/30th of the human genome [260], shows a remarkable conservation (approx. 40%) with that of humans [68], and is easily manipulatable by several methods, such as forward genetic screen using chemical mutagenesis [63], reverse genetic screen using RNA‐interference (RNAi) [261], or the relatively newly developed precise manipulation by techniques, such as CRISPR/Cas9 [262]. Similarly, enhancer and suppressor screens can be carried out in disease models to identify additional genes that modulate the phenotype of interest (reviewed in [263, 264] and has provided insights into genetic interactions and pathway crosstalk.

Regarding TDP‐43, functional alterations in genes in transgenic *C. elegans* models have yielded several disease‐interacting partners. In the YFP‐tagged TDP‐43 transgenic model that displays temperature‐dependent neurotoxicity, an RNAi screen targeted against components of the protein quality‐control system identified heat‐shock factor (HSF‐1) (see Table 5) as a major player whose absence resulted in a worsened neurotoxicity, particularly in worms overexpressing the YFP‐TDP‐C25 fragment [143]. Using a similar RNAi‐mediated one gene depletion approach, a potential role of AMPK in TDP‐43 induced toxicity was investigated using worms deficient in *aak‐2* (*C. elegans* AMPK α2 orthologue), for reasons that some ALS patients possess metabolic defects [265] and AMPK being a well‐known metabolite sensor [266]. Only mutant TDP‐43 (not wild‐type) over‐expressing worms benefitted from an absence/reduction of the *C. elegans* AMPK orthologue *aak‐2* by displaying an improved motor function. However, the neurodegeneration persevered in the *aak‐2* deficient background [267].

**Table 5:** Summary of the suppressor/chemical screen in *C. elegans* TDP‐43 models.

TDP‐43 model	Genetic modulator	Compound modulator
YFP‐TDP‐C25	*hsf‐1* [143] *daf‐2* (insulin/IGF1R) [143]	PROTACs [311]
TDP‐43(M337V)	*aak‐2* (AMPK) [267] *cdc‐2* (CDC‐7) [268] C55B7.10 (tau tubulin Kinases) [268] TTBK1/2 (H05L14.1) [273] PRKD2/3 (*dkf‐2*) [273]	PHA767491 [268]
TDP‐43 (WT) TDP‐43(A315T) TDP‐43 (M337V)	*tax‐1* (calcineurin) [279] *rad‐23* (ZK20.3) *ufd‐3* (C05C10.6) *ubxn‐4* (UBXN‐4) *ufd‐2* (T05H10.5) [277]	
TDP‐43 (A315T) TDP‐43 (M337V)	Genome‐wide screen, multiple candidates identified including *hse‐5* (glucuronic acid epimerase GLCE) [281]	
TDP‐43(A315T)	*aly‐2/3* (ALYREF) [285]	Lithium chloride, methylene blue, riluzole [291] Glucose [293] Salubrinal, guanabenz and phenazine [292] 11 compounds tested of which resveratrol, rolipram, reserpine, trolox, propyl gallate, and ethosuximide reduced mutant TDP‐43 neuronal toxicity [306] SB203580 [207] Maple syrup [296] Ethosuximide‐based compounds identified α‐methyl‐α‐phenylsuccinimide (MPS) [308] High throughput screen (3,765 molecules) identified TRVA242 [303] ATP‐competitive and 6‐mercaptopurine‐based CDC7 inhibitors [303] High throughput screen of 3,850 small molecules identified 13 neuroleptic compounds: mianserin, amoxapine, nicergoline, kawain, pimethixene, methiotepin, octoclopethin, flupentixol, chlorprothixene clozapine, pizotifen, cyproheptadine, and Pimozide, with Pimozide as the most potent [297], currently in clinical trial [300] HA‐114 from the probiotic bacterial strain *Lacticaseibacillus rhamnosus* [299], currently in clinical trial [301] Cell‐line based high‐throughput library screen of 1,500 molecules identified the alkaloid lycorine, proving effective in *C. elegans* and mouse TDP‐43 models [309]
SUP‐46 TDP‐1‐mCherry	*ubc‐9* [203]	
TDP‐43(WT)	*egl‐19(ad695)*, *slo‐1(js118)* [222]	Arecoline, phorbol esters, serotonin antagonists (mianserin, methiothepin), L‐type calcium channel agonist (nefiracetam), potassium channel blockers (iberiotoxin, 4‐aminopyridine) [222]

Since a causal relationship between the pathological phosphorylation and neurotoxicity was observed in mutant TDP‐43 over‐expressing worms [192], an RNAi screen targeting 95% of *C. elegans* kinases was carried out to identify kinases with a potential role in the pathological phosphorylation and the observed neurotoxicity [268]. This resulted in several hits, out of which *cdc‐7* and *C55B7.10* loss reduced the pathological TDP‐43 phosphorylation (pS409/410) [268]. Human CDC7 shows similarity with the only *C. elegans cdc‐7* kinase, as revealed by molecular phylogenetics [268] and is known to play multiple roles in cell cycle pathways [269, 270, 271, 272]. It directly phosphorylates TDP‐43, and its loss reduces neurodegeneration and partially restores the motor function associated with TDP‐43 over‐expression [268]. Two other kinases were also identified namely *H05L14.1* and *dkf‐2*. Phylogenetic analysis revealed that H05L14.1 is identical to human tubulin kinases TTBK1 and TTBK2 [273], which are involved in disease‐relevant pathways in multiple neurodegenerative diseases, reviewed in [274]. Both TTBK1 and 2 promoted S409/410 pathological phosphorylation via a direct interaction with TDP‐43 [273]. Despite both TTBK1 and TTBK2 interacting directly with TDP‐43 *in vitro*, overexpression of the catalytic domain of human TTBK1 but not TTBK2 increased the phospho‐ and insoluble TDP‐43 levels and the associated motor dysfunction in *C. elegans* [275]. *dkf‐2*, that was found to share significant homology in the kinase domain with human protein kinase D2 and D3 (PRKD2/3), on the other hand, showed no direct interaction with TDP‐43. Despite the lack of direct interaction, *dkf‐2* loss also improved the motor function of transgenic worms overexpressing TDP‐43 [273]. Similarly, candidate‐based knockdown of the components of ER‐associated degradation (ERAD), a multistep process that removes misfolded proteins from the ER through the 26S proteasome [276], by Kalb and colleagues in mutant TDP‐43M337V *C. elegans* identified RAD‐23, loss of which ameliorated the toxicity due to mutant TDP‐43 and SOD‐1 not only in transgenic *C. elegans*, but also in mammalian neurons [277]. Correspondingly, spinal cord tissue samples from human ALS patients showed increased levels and mislocalisation of RAD‐23 within motor neurons [277]. Subsequent work by the same group utilised a cellular model to look at the proteome remodeling due to RAD‐23 loss and identified deubiquitinase USP13 as a strong modifier of TDP‐43 aggregation and cytotoxicity, with reproducible results in HEK293 cell model, primary rat neurons and *C. elegans* ALS models [278].

In a different study, a yeast two‐hybrid screen based on TDP‐43 C‐terminus that harbours the pathological phosphorylation sites identified phosphatase Ca^2+^/calmodulin‐dependent calcineurin as a binding partner of both wild‐type and the familial ALS mutant TDP‐43 variants [279]. Using *tax‐1* and *cnb‐1* mutant worms deficient in catalytic and regulatory subunits of the *C. elegans* calcineurin orthologue respectively, both phospho‐ and the insoluble TDP‐43 levels increased in TDP‐43 over‐expressing worms, which was subsequently associated with an exacerbated motor dysfunction and increased neurodegeneration [279]. Upon immunohistochemical examination of the postmortem neurons of FTLD‐TDP and ALS patient brains, both the groups reported a clear association of the individual candidates, i.e. TTBK1/2 [273] and calcineurin [279], with the phospho‐TDP‐positive inclusions. It is worth mentioning that while a calcineurin loss acted synergistically to the TDP‐43‐induced toxicity in worms [279], an opposite effect was reported in *C. elegans* lines transgenic for proteins implicated in other neurodegenerative diseases where its loss, for example, ameliorated the tau‐induced neurotoxicity [205]. The caveat though is that the apparent toxicity in this tau transgenic model was partially attributed to Ca^2+^ dysregulation [205], and not due to insoluble protein aggregates [280].

In a new RNAi screen, this time targeting 86% of *C. elegans* genome, candidate genes with homologues in humans and those that suppressed the motor dysfunction without affecting the total TDP‐43 or phosphor‐TDP‐43 levels were selected and show involvement in multiple biological pathways [281]. Among these, *hse‐5* and its human homologue heparan sulfate‐modifying enzyme glucuronic acid epimerase (GLCE) were studied, and depletion of both reduced the TDP‐43‐associated toxicity in the respective set‐ups, i.e. *C. elegans* and cellular TDP‐43 models [281]. And immunohistochemical examination of FTLD‐TDP and ALS postmortem brain tissue showed reduced GLCE expression pattern in phospho‐TDP‐43‐positive inclusions [281].

In yet another effort for a continued search for novel pathways that participate in these diseases, genetic interaction between ALYREF, a component of mRNA nuclear export complex [282], whose levels in ALS patient motor neurons with TDP‐43 pathology are altered [283], and an interaction partner of MSUT‐2, a suppressor identified in a genetic screen in a *C. elegans* tauopathy model [284], was tested. Depletion of *C. elegans* ALYREF orthologues *aly‐2* and *aly‐3* improved the motor function without affecting the total‐ or phospho‐TDP‐43 protein or its mRNA levels [285]. This is in sharp contrast to a drosophila TDP‐43 [283] or a *C. elegans* tauopathy model [285], where depletion of respective ALYREF homologues *Ref* and *aly* suppressed the mRNA and total protein levels of the transgenes (TDP‐43 and tau respectively). Overall, the genetic tractability and conservation of disease‐relevant pathways makes *C. elegans* invaluable for uncovering genetic and molecular mechanisms underlying neurodegeneration in TDP‐43 proteinopathies.

### Chemical modulators

Since the implication of TDP‐43 in the pathogenesis of ALS and FTLD, multiple aspects of TDP‐43 proteinopathies, such as TDP‐43 expression levels, altered intracellular distribution, post‐translational modifications, truncation, aggregation, degradation, etc. have been targeted for candidate drug screening (reviewed in [286]), with an aim to ultimately put them to the clinical test for their ability to halt or delay the progression of these debilitating diseases in human patients. However, the overwhelming costs associated with high‐throughput drug screening demands initial screening using lower animal models before their validation and optimisation in mammalian models.

*Caenorhabditis elegans* has long been a model organism of choice for cost‐effective and rapid high‐throughput drug screening. *C. elegans* offers a range of behaviours that can be modulated by drugs like locomotion, feeding, reproduction, and response to environmental cues, providing valuable information about the effects of drugs on neural function and behaviour, as well as potential therapeutic applications for neurological disorders. Drug effects on fundamental biological processes shared with higher animals including humans can be studied in *C. elegans*, providing insights into potential drug mechanisms and toxicity profiles. The genetic manipulability that enables researchers to create transgenic or mutant strains with specific genetic backgrounds, allows for the investigation of drug interactions with specific genes or pathways of interest. For example, specific genes can be silenced using RNAi and then screened for drugs that modulate the resulting phenotype. Likewise, disease models can be used to screen compounds that rescue or alleviate disease phenotypes [287, 288, 289], leading to the identification of potential drug candidates for further investigation. That stated, obstacles that impede drug assays in *C. elegans* do exist but have been largely addressed [263]. Additionally, the recent advent of various automated platforms and microfluidic devices [290] have facilitated the streamlining of drug screening processes, enabling the swift testing of thousands of compounds for their effects on *C. elegans* physiology, behaviour, or disease phenotypes.

Among the *C. elegans* transgenic models discussed previously, several have been employed to identify compounds capable of altering the disease phenotype associated with TDP‐43 over‐expression (see Table 5). In one such example, out of the three compounds that were tested in *C. elegans* and zebra fish TDP‐43 models, namely lithium chloride, methylene blue and the FDA approved drug for ALS patients riluzole, methylene blue mitigated the TDP‐43‐induced neurotoxic effects in both the models [291], in a mechanism involving endoplasmic reticulum (ER) stress response [292]. In a follow‐up study, three more compounds namely salubrinal, guanabenz and phenazine were identified which mitigated the TDP‐43‐induced paralysis, neurodegeneration, and oxidative stress in *C. elegans* through a mechanism also involving the ER stress response, such as methylene blue [292].

Parker and colleagues investigated the role of metabolism in TDP‐43‐mediated neurotoxicity in transgenic *C. elegans* [293]. Calorie restriction that promotes longevity and mitigates the paralysis in a *C. elegans* transgenic A‐beta_1‐42_ model [294, 295], had no role in the TDP‐43‐mediated neurotoxicity [293]. Contrary to these studies, glucose enrichment mitigated the paralysis and neurodegeneration, and reduced the insoluble mutant TDP‐43 fraction in the mutant but not the wild‐type TDP‐43 transgenic *C. elegans*, and these effects required glucose to be metabolised, as well as the activities of DAF‐16 and HSF‐1 [293]. In line with these findings, maple syrup that is rich in numerous active compounds including sugars and phenol was found to be neuroprotective in mutant TDP‐43A315T transgenic *C. elegans* in a DAF‐16‐dependent pathway [296]. Expanding on these metabolism‐based therapeutic strategies, a high‐throughput screen of 3,850 small molecules in *C. elegans* TDP‐43A315T model identified 13 neuroleptic compounds [297]. Following validation in a zebrafish model expressing analogous mutant TDP‐43 [298], pimozide emerged as the most potent, capable of alleviating neurodegeneration and improving the motor function by restoring neuromuscular junction activity through T‐type Ca^2+^ channels [297]. Similarly, a probiotic screen pinpointed *Lacticaseibacillus rhamnosus* HA‐114 as a strain capable of mitigating motor deficits and neurodegeneration in the *C. elegans* TDP‐43A315T ALS model, and also in Huntington's disease model through modulation of lipid metabolism [299]. Based on these preclinical findings, both Pimozide and HA‐114 progressed to clinical trials. While a phase 2 clinical trial [300] evaluated the safety, tolerability, and efficacy of pimozide, larger studies are expected to confirm its efficacy against ALS. As for HA‐114, clinical trials exploring whether targeted probiotic supplementation can beneficially modify lipidomic profiles in Amyotrophic Lateral Sclerosis‐Frontotemporal Dementia Spectrum Disorder (ALS‐FTDSD) patients is underway [301]. These two represent classical examples of potential therapeutic intervention against ALS through translational research built upon *C. elegans* work.

In yet another high‐throughput approach, Bose et al. used a transgenic *C. elegans* TDP‐43 model to screen a compound library of 3765 molecules based on the repurposed neuroleptic drug pimozide, and the hits were eventually confirmed and validated in higher animal models of ALS in relation to zebrafish and mice, respectively. TRVA242 proved to be the most efficacious and mitigated the neurotoxicity in multiple animal models of ALS [302].

Moreover, an indirect approach targeting the genetic suppressors identified through genetic screens in transgenic *C. elegans* TDP‐43 models has also been implemented. One such example is the kinase inhibitor PHA767491 targeted against the previously identified genetic suppressor CDC7 [268] that reversed the pathological phosphorylation of TDP‐43 and reduced the resulting neurodegeneration [268]. In line with this, Rojas‐Prats et al. designed a new class of ATP‐competitive and mercaptopurine‐based CDC7 inhibitors that are specific to CDC7 and brain permeable [303], unlike PHA767491 that is blood‐brain barrier impermeable and non‐specific [268]. This new class of CDC7 inhibitors were eventually tested in multiple TDP‐43 models, such as cellular, *C. elegans* and mice, faithfully yielding the desired results, in relation to reduced TDP‐43 phosphorylation [303]. Similarly, a small molecule targeted against the downstream TIR‐1 pathway kinase PMK‐1/p38 MAPK reduced the paralysis and neurodegeneration in mutant TDP‐43 transgenic worms [207].

Ethosuximide, the anti‐epileptic drug with a proven efficacy in multiple *C. elegans* disease models [304, 305], also mitigated the neurotoxicity in TDP‐43 transgenic *C. elegans* via a DAF‐16 dependent mechanism [306], similar to its effects on longevity [304, 307]. Besides ethosuximide, other longevity‐promoting compounds like resveratrol, rolipram, reserpine, trolox and propyl gallate were found to mitigate the mutant TDP‐43‐induced neurotoxicity in *C. elegans*, with resveratrol requiring *daf‐16* and *sir‐2.1* for neuroprotection [306]. These studies prompted another independent group to design compounds based on ethosuximide that led to the identification of α‐Methyl‐α‐phenylsuccinimide as a potent inhibitor of neurotoxicity and neurodegeneration associated with mutant TDP‐43 overexpression in *C. elegans* via a similar mechanism to ethosuximide that required the FOXO transcription factor DAF‐16 [308]. A classic example of designing a mechanism‐based therapy was provided in yet another screen in TDP‐43 transgenic worms presenting along with other phenotypes the impaired coupling between neuronal excitation and secretion, albeit with a functional synaptic priming and fusion machinery [222], an L‐type calcium channel agonist (nefiracetam) and two potassium channel blockers (iberiotoxin and 4‐aminopyrdine), which were identified as positive hits that improved the locomotion [222]. The same was also achieved by the genetic manipulation of targets of two of these compounds, L‐type Ca^2+^ channel (*egl‐19‐ad695*) and Ca^2+^‐dependent SLO‐1 K^+^ channel (*slo‐1‐js118*) [222]. Similarly, a mutant TDP‐43A152T cell‐line‐based high‐throughput screening from a natural product library of 1,500 compounds identified a naturally occurring alkaloid lycorine as a potent inhibitor of cytotoxicity [309]. Lycorine reduced the TDP‐43 burden, both by targeting its synthesis and promoting its degradation by a ubiquitin‐proteasome system (UPS), and subsequently proved equally effective in in vivo TDP‐43 models, such as *C. elegans* and mouse [309].

Other strategies involve harnessing the cellular machinery responsible for protein turnover using for example proteolysis‐targeting chimeras (PROTACs), a novel class of heterobifunctional small‐molecule degraders consisting of an E3 ligase, recruiting moiety and a binding ligand specific to the target protein [310]. Fang and colleagues designed PROTACs for forced ubiquitylation of TDP‐43 and subsequent degradation by proteasome. PROTACs selectively degraded misfolded C‐terminal TDP‐43 in cellular and *C. elegans* models, reducing the aggregated TDP‐43 burden and the associated neurotoxicity [311]. Recent advances in our understanding of some of the cellular pathways that underlie targeted degradation by PROTACs [312] should allow chemical compounds to be designed that will facilitate PROTAC‐induced degradation of disease proteins including TDP‐43.

## Conclusion

Neurodegenerative disorders are complex, multisystemic disorders with often unknown underlying causes, requiring therefore identification of modifiable factors of neurodegeneration to guide effective prevention and therapeutic success. Transgenic worms engineered to express full‐length or truncated TDP‐43 in neurons or muscle cells have recapitulated key if not all, pathological features observed in human diseases, including protein condensation, aggregation, neurodegeneration, and motor deficits, and have provided valuable insights into the pathophysiological mechanisms underlying TDP‐proteinopathies. To conclude, despite its inherent limitations that necessitate the use of vertebrate models for studying these multi‐organ, immune‐mediated, and behaviourally complex human diseases, *C. elegans* remains a powerful first‐pass model for genetic, developmental, and basic molecular processes and signaling, and its short lifespan allows for studying ageing phenotypes more expediently than other systems.

## Future directions

Several future perspectives can be envisioned by leveraging the genetic tractability and short lifespan of *C. elegans* to screen large libraries of compounds and genetic mutations to uncover potential therapeutic targets and disease modifiers. Another important avenue for future research which worm models are ideally suited to is the exploration of cell‐type‐based selective vulnerability and resilience of neurons (that have received the most attention so far), as well as the contribution of underrepresented non‐neuronal cells to TDP‐43 pathology. These studies have the potential to expand the toolbox for manipulating biological processes to identify novel cell‐type‐specific therapeutic strategies. RNA metabolism in TDP‐43 pathology is another aspect that can be addressed through RNAi or CRISPR/Cas9‐mediated editing of genes involved in RNA processing, to understand its impact on neurodegeneration. Similarly, TDP‐43 aggregation and clearance mechanisms, such as autophagy, proteasome and protein quality control can be investigated using fluorescently tagged TDP‐43 in *C. elegans* with its transparent body that allows live imaging. Also, worm models hold immense promises for a deeper understanding of the dynamic changes in gene expression, protein interactions, and cellular processes by applying techniques such as advanced imaging, RNA sequencing, and proteomics to unravel the complex molecular networks and pathways involved in TDP‐43 proteinopathies.

Finally, with modern genetic engineering technologies, next‐generation approaches need to be increasingly employed to enhance the fidelity of neurodegenerative disease models. These include bi‐/multi‐genic models that incorporate additional disease‐associated genes, single‐copy insertions to mimic endogenous expression levels, humanised models that express orthologous human genes in place of their native counterparts, and inducible transgene systems that allow temporal and tissue‐specific control of gene expression. Some of these advanced approaches, like the optogenetic TDP‐43 modulation in *C. elegans* that resulted in light‐induced ALS pathology in relation to motor impairment, reduced lifespan and TDP‐43 inclusions in cell bodies and neurites, which were otherwise absent [313], are being implemented to refine disease modeling, offering more physiologically relevant models for studying disease mechanisms and therapeutic interventions.

## Conflict of interest

Dr. Steinhoff has performed consultancy services for Pfizer, Sanofi, Regeneron, Lilly, Novartis, Galderma, Leo, Merck, Avon, Pierre‐Fabre, L'Oreal, BMS, Maruho, Toray, Mitsubishi, Maruho, Kiniksa, ZymoGenetics, and Almirall, for which he received compensation; he served on the advisory board for Pfizer, Novartis, Galderma, Leo, Avon, Pierre‐Fabre, L'Oreal, BMS, Maruho, Toray, Mitsubishi, Maruho, ZymoGenetics, Almirall; his research was supported by Pfizer, Novartis, Galderma, Leo, Avon, Pierre‐Fabre, L'Oreal, BMS, Maruho, Toray, Mitsubishi, Maruho, ZymoGenetics and Almirall. None of the other authors have any other conflicts of interest to report.

## Author contributions

GJP: conceptualisation, research and writing–original draft, writing–review and editing. JB: writing–review and editing. MAA: writing–review and editing. AO: writing–review and editing. RJE: writing–review and editing. BCK: writing–review and editing. EM: writing–review and editing. MS: funding acquisition, writing–review and editing.
